# PLPP/CIN regulates bidirectional synaptic plasticity via GluN2A interaction with postsynaptic proteins

**DOI:** 10.1038/srep26576

**Published:** 2016-05-23

**Authors:** Ji-Eun Kim, Yeon-Joo Kim, Duk-Shin Lee, Ji Yang Kim, Ah-Reum Ko, Hye-Won Hyun, Min Ju Kim, Tae-Cheon Kang

**Affiliations:** 1 Department of Anatomy and Neurobiology, Institute of Epilepsy Research, College of Medicine, Hallym University, Chuncheon 200-702, South Korea

## Abstract

Dendritic spines are dynamic structures whose efficacies and morphologies are modulated by activity-dependent synaptic plasticity. The actin cytoskeleton plays an important role in stabilization and structural modification of spines. However, the regulatory mechanism by which it alters the plasticity threshold remains elusive. Here, we demonstrate the role of pyridoxal-5′-phosphate phosphatase/chronophin (PLPP/CIN), one of the cofilin-mediated F-actin regulators, in modulating synaptic plasticity *in vivo*. PLPP/CIN transgenic (Tg) mice had immature spines with small heads, while PLPP/CIN knockout (KO) mice had gigantic spines. Furthermore, PLPP/CIN Tg mice exhibited enhanced synaptic plasticity, but KO mice showed abnormal synaptic plasticity. The PLPP/CIN-induced alterations in synaptic plasticity were consistent with the acquisition and the recall capacity of spatial learning. PLPP/CIN also enhanced N-methyl-D-aspartate receptor (GluN) functionality by regulating the coupling of GluN2A with interacting proteins, particularly postsynaptic density-95 (PSD95). Therefore, these results suggest that PLPP/CIN may be an important factor for regulating the plasticity threshold.

Long-term potentiation (LTP) and its counterpart, long-term depression (LTD), represent synaptic plasticity, which involves learning, memory and experience-dependent development of cortical circuitry^1^. LTP is a long-lasting increase in synaptic efficacy, which requires the filamentous actin (F-actin) polymerization, Ca^2+^/calmodulin-dependent protein kinase II (CaMKII) activation and the translocation of α-amino-3-hydroxy-5-methyl-4-isoxazolepropionic acid receptor (GluA) to the synapses^2^,^3^. In contrast, LTD is an activity-dependent reduction in the efficacy of neuronal synapses through F-actin destabilization and GluA internalization, which is mediated by phosphatases including calcineurin (CN)^4^. Both LTP and LTD are initiated by the entry of Ca^2+^ via excitatory receptors including N-methyl-D-aspartate receptor (GluN)^5^. Bienenstock *et al*.^6^ suggested a “sliding threshold” model for LTP and LTD induction. Briefly, high level of postsynaptic [Ca^2+^]_i_ induced by a previous synaptic activation would increase the possibility of input to elicit LTD. Conversely, low level of postsynaptic [Ca^2+^]_i_ would favor the induction of LTP. However, the molecular mechanisms regulating the threshold of synaptic plasticity that are necessary to maintain synaptic strength and plasticity, are poorly understood.

F-actin is important for the structural modification of spines during synaptic plasticity, which is regulated by cofilin^7^,^8^,^9^,^10^. Pyridoxal-5′-phosphate phosphatase/chronophin (PLPP/CIN) activates cofilin activity by Ser-3 site dephosphorylation^11^, whereas LIM kinases (LIMK1 and -2)-mediated phosphorylation inhibits its activity^12^,^13^. F-actin dynamics are considered as a secondary phenomena following synaptic plasticity induction; however, it is possible that F-actin itself regulates synaptic plasticity via modulation of GluN activation^14^,^15^. Some research has shown that abnormal actin dynamics inhibit LTP and LTD inductions^4^,^16^. Therefore, it remains to be determined whether F-actin changes the threshold of synaptic plasticity and the molecular mechanisms involved. In order to address these questions, we have generated PLPP/CIN transgenic (Tg) mice, as well as PLPP/CIN knockout (KO) mice. Here, we demonstrate that PLPP/CIN Tg mice had immature spines with small heads, while KO mice had abnormal gigantic spines. Unexpectedly, PLPP/CIN Tg mice showed the enhancement of synaptic plasticity, but KO mice showed abnormal synaptic plasticity. PLPP/CIN also affected spatial learning. Furthermore, PLPP/CIN modulated the coupling of GluN subunit 2A (GluN2A) with interacting proteins, particularly postsynaptic density-95 (PSD95). Therefore, PLPP/CIN is a potentially important factor for regulating the plasticity threshold via F-actin-mediated GluN activity.

## Results

### PLPP/CIN-mediated F-actin depolymerization regulates dendritic spine morphology

**Figure 1:**
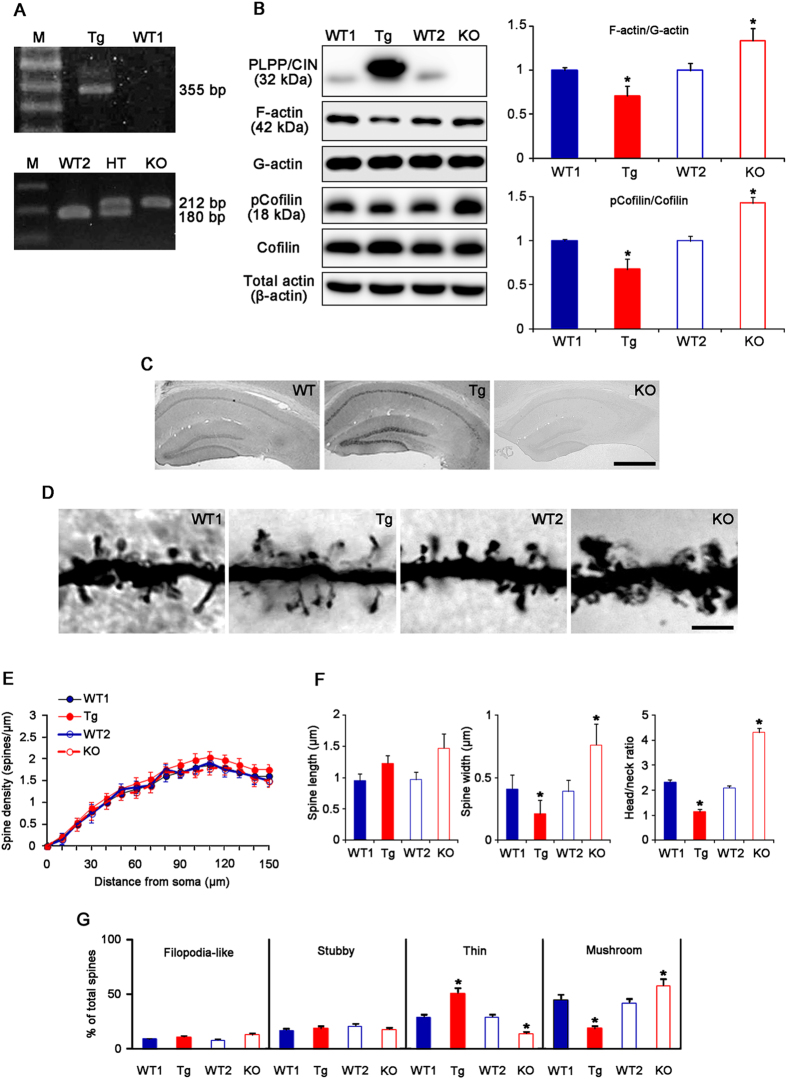
Profiles of PLPP/CIN Tg and PLPP/CIN KO mice. (**A**) Genotyping analysis of PLPP/CIN Tg (upper) and KO (lower) mice. Genomic DNA was isolated from the tail, and amplified using specific primers. The PCR product was analyzed by gel electrophoresis. An 355 bp fragment for Human *PLPP/CIN* is detected only in Tg mice. An 180 bp fragment for WT specific band is detected in WT mice. An 212 bp fragment for mutant specific band is detected in KO mice. Both WT specific and mutant specific bands are detected in hetero (*PLPP/CIN*^+/−^) animal. (**B**) Analyses of F-actin content and cofilin activity. As compared to WT animals, PLPP/CIN protein expression is increased in the hippocampus of Tg mice, but absent in that of KO mice. As compared to WT animals, both F-actin/G-actin ratio and pCofilin/cofilin ratio are lower in Tg mice, but was higher in KO mice (*p < 0.05; n = 7, respectively). Error bars in graphs indicates SEM. (**C**) Localization of PLPP/CIN in the hippocampus. In Tg mouse, PLPP/CIN protein expression increases in CA1 pyramidal neurons as well as dentate granule cells, as compared to WT mouse. In KO mice, PLPP/CIN expression is absent in the hippocampus. Bar = 200 μm. (**D**) Representative spine morphology of dentate granule cells. Bar = 5 μm. (**E**) Spine density in dendrites of dentate granule cells. (*p < 0.05; n = 7, respectively). Error bars in graphs indicates SD. (**F**) Spine length, spine width and head/neck ratios of dentate granule cells. (*p < 0.05 vs. WT animals; n = 7, respectively). Error bars in graphs indicates SD. (**G**) Percentage of categories of spine classification in dentate granule cells. (*p < 0.05 vs. WT animals; n = 7, respectively). Error bars in graphs indicates SEM.

We generated and bred PLPP/CIN Tg mice overexpressing human PLPP/CIN on a C57BL/6J background and PLPP/CIN KO mice on a 129/SvEv-C57BL/6J background (Fig. 1A and Supplementary Fig. 1). Therefore, C57BL/6J (wild-type mice 1, WT1) and 129/SvEv-C57BL/6J mice (wild-type mice 2, WT2) were used as controls for Tg and KO mice, respectively. PLPP/CIN was constitutively expressed in the hippocampus of Tg mice, but absent in that of KO mice (Fig. 1B). As compared to WT animals, F-actin content was lower in Tg mice, but was higher in KO mice. In addition, phospho-Cofilin (pCofilin)/Cofilin ratio was decreased in Tg animals, but increased in the KO animals (*n* = 7, respectively, *p* < 0.05 vs. WT animals, Fig. 1B).

PLPP/CIN was overexpressed in CA1 pyramidal neurons and dentate granule neurons in Tg mice (Fig. 1C). Unlike the rat hippocampus^17^,^18^, astroglial PLPP/CIN expression level was very low, but not absent in the WT animals. In addition, there was no difference in astroglial PLPP/CIN expression between WT1 and Tg mice (Supplementary Fig. 2). Therefore, the low astroglial PLPP/CIN expression in the mouse hippocampus could exclude the possibility that astroglial dysfunction would evoke alterations in synaptic functions.

PLPP/CIN overexpression or deletion did not affect the dendrite length and the total number of branch points per dentate granule cells (Supplementary Fig. 3A). However, spine shape of dentate granule cells was clearly altered in Tg and KO mice, despite the lack of difference in spine density (*n* = 7, respectively, Fig. 1D,E). Average of spine length in the WT1 and WT2 mice were 0.95 and 0.97 μm, while those in the Tg and KO mice were 1.23 and 1.47 μm, respectively (Fig. 1F). Average spine width in the WT1 and WT2 mice were 0.41 and 0.39 μm, but those in the Tg and KO mice were 0.21 and 0.76 μm, respectively (Fig. 1F). Therefore, spines of the dentate granule cells in the WT mice displayed thin necks and relatively large heads, with a head/neck ratio of >2. In contrast, the spines in the Tg mice showed thin necks with very small heads (head/neck ratio: 1.13). KO mice showed gigantic spines, with a head/neck ratio of >4 (Fig. 1D,F). As compared to WT animals, the fraction of thin spines in total spines was increased, but that of mushroom spines was decreased in Tg mice (Fig. 1G). The fraction of mushroom spines in the total spines was increased, while that of thin spines was decreased in KO mice (Fig. 1G). There was no difference in the fraction of filopodia-like and stubby spines in total spines among four groups. These alterations in the spine morphology were similarly observed in CA1 neurons (Supplementary Fig. 3B). Therefore, our findings indicate a potential role for PLPP/CIN in the regulation of spine morphology.

### PLPP/CIN expression does not affect GABAergic transmission and short-term presynaptic plasticity

To investigate the physiological profiles of Tg and KO mice, we compared the paired-pulse responses of the dentate gyrus. There were no differences in the input-output (IO) curves among four groups (*n* = 10, respectively, *p* < 0.05, Supplementary Fig. 4A). All animals showed paired-pulse depressions at 20- and 30-ms interstimulus intervals, and paired-pulse facilitations at 70- and 150-ms interstimulus intervals (*n* = 10, respectively, Supplementary Fig. 4B). There were no differences in the normalized population spike amplitude ratios (second population spike amplitude/first population spike amplitude) at any interstimulus interval among the 4 groups (*n* = 10, respectively, Supplementary Fig. 4C). Paired-pulse inhibition and paired-pulse facilitation are used as indicators of GABAergic transmission and presynaptic probability of glutamate release, respectively^19^. Hence, our findings indicate that PLPP/CIN may not affect GABAergic neurotransmission and a short-term presynaptic plasticity (the degree of paired-pulse facilitation).

### PLPP/CIN deletion disrupts LTP induction, but its overexpression enhances LTP

**Figure 2:**
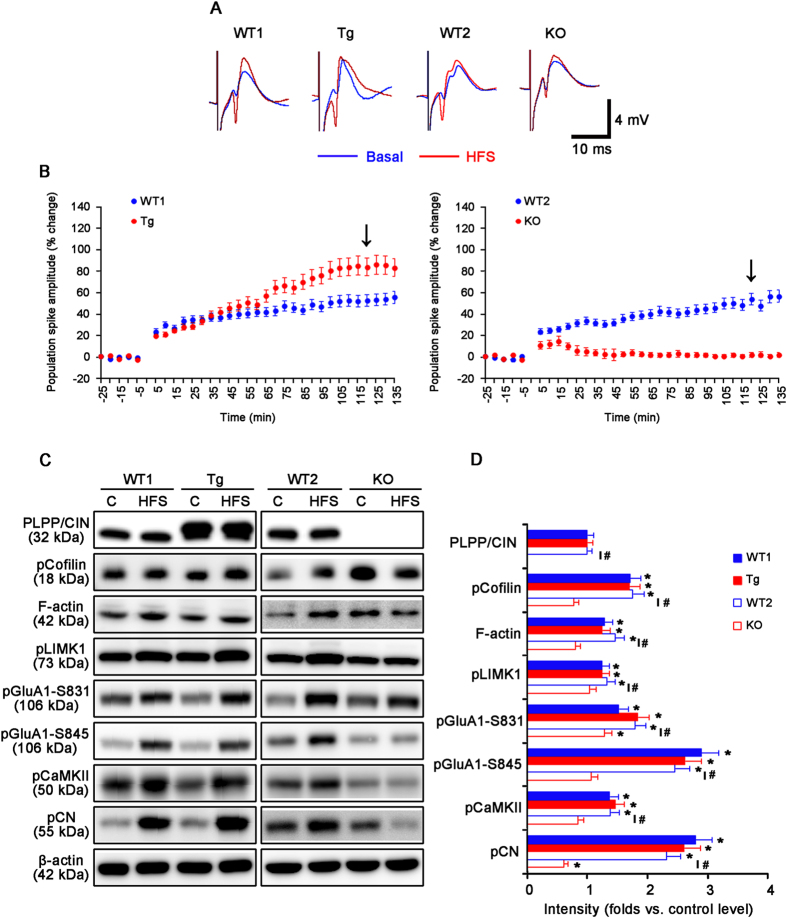
Profiles of LTP induction induced by HFS. (**A**) Representative traces of LTP induction in the dentate gyrus. (**B**) The quantitative analyses of the LTP induction. As compared to WT animals, Tg mice show enhanced LTP induction, while KO mice exhibit disrupted LTP induction (p < 0.05; n = 10, respectively). Error bars in graphs indicates SEM. Arrows indicates the time point of representative traces. (**C**,**D**) Western blot analysis of the efficacy of LTP induction. HFS increases F-actin level and phosphorylation levels of cofilin, LIMK1, GluA1, CaMKII and CN in WT and Tg mice. In KO mice, HFS increases pGluA1-S831 level, but reduced pCN level (*p < 0.05 vs. control level, ^#^*p* < 0.05 vs. WT animals; n = 10, respectively). Error bars in graphs indicates SEM.

Next, we investigated whether PLPP/CIN affects LTP induction by high frequency stimulus (HFS). HFS produces a significant LTP in WT1, WT2 and Tg mice (*n* = 10, respectively, Fig. 2A,B and Supplementary Fig. 5A). The efficacy of LTP in Tg mice was higher than that in WT1 mice (*n* = 10, respectively, *P* < 0.05 vs. WT1 animals, Fig. 2A,B and Supplementary Fig. 5A). HFS did not evoke sustained LTP in KO mice (*n* = 10, respectively, *P* < 0.05 vs. WT2 animals, Fig. 2A,B and Supplementary Fig. 5A). There was no difference in the basal expression levels of cofilin, LIMK1, GluA1, CaMKII and CN among all experimental groups; and furthermore, HFS did not affect their expression levels including PLPP/CIN (Fig. 2C,D and Supplementary Fig. 6). HFS increased F-actin level accompanied by enhanced cofilin and LIMK1 phosphorylation in WT1 and Tg mice (*n* = 10, respectively; *P* < 0.05 vs. control level, Fig. 2C,D). There was no difference in F-actin, pCofilin and pLIMK1 levels between WT1 and Tg mice. HFS also increased F-actin, pCofilin and pLIMK1 levels in WT2 mice (*P* < 0.05 vs. control level), but not KO mice (*n* = 10, respectively; *P* < 0.05 vs. WT2 animals, Fig. 2C,D). The regulation of GluA1 phosphorylation by CaMKII and CN plays an important role in synaptic plasticity^20^,^21^,^22^,^23^. In addition, pCaMKII represents increased CaMKII activity, and pCN indicates decreased CN activity^24^,^25^. Thus, we evaluated pGluA1, pCaMKII and pCN levels in response to HFS. HFS increased pGluA1-S831 and -S845 levels in WT1 and Tg mice (*n* = 10, respectively; *P* < 0.05 vs. control level, Fig. 2C,D). No difference in pGluA1 level was detected between WT1 and Tg mice. Similarly, HFS enhanced both pGluA1-S831 and -S845 levels in WT2 animals (*P* < 0.05 vs. control level). HFS increased only the pGluA1-S831 level in KO mice (*P* < 0.05 vs. control level), which was lower than that in WT2 mice (*P* < 0.05, Fig. 2C,D). Furthermore, HFS increased pCaMKII and pCN levels in WT1, Tg and WT2 mice (*n* = 10, respectively; *P* < 0.05 vs. control level, Fig. 2C,D). However, HFS did not affect pCaMKII level, and reduced pCN level in KO mice (*n* = 10, respectively; *P* < 0.05 vs. WT2 animals, Fig. 2C,D). Therefore, our findings suggest that PLPP/CIN may be essential for LTP induction.

### PLPP/CIN overexpression enhances LTD, while its deletion perturbs the maintenance of LTD

**Figure 3:**
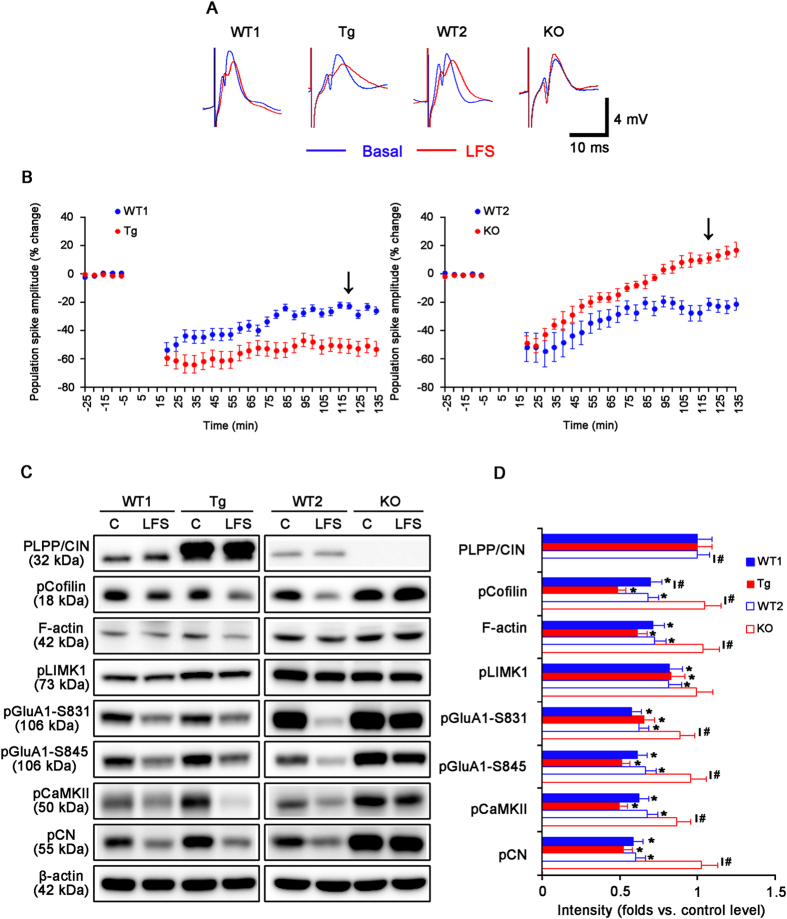
Characteristics of LTD induction induced by LFS. (**A**) Representative traces of LTD induction in the dentate gyrus. (**B**) The quantitative analyses of the LTD induction. As compared to WT animals, Tg mice show enhanced LTD induction, while KO mice exhibit short-term depression (p < 0.05; n = 10, respectively). Error bars in graphs indicates SEM. Arrows indicates the time point of representative traces. (**C**,**D**) Western blot analysis of the efficacy of LTD induction. LFS decreases F-actin level and phosphorylation levels of cofilin, LIMK1, GluA1, CaMKII and CN in WT and Tg mice. In KO mice, LFS does not affect protein phosphorylation level (*p < 0.05 vs. control level, ^#^*p* < 0.05 vs. WT animals; n = 10, respectively). Error bars in graphs indicates SEM.

We also explored the influence of PLPP/CIN on LTD induction by low frequency stimulus (LFS). LFS reliably induced a robust LTD in WT1, WT2 and Tg mice (*n* = 10, respectively; Fig. 3A,B). The efficacy of LTD in Tg mice was higher than that in WT1 mice (*n* = 10, respectively; *P* < 0.05 vs. WT2 animals, Fig. 3A,B). In KO mice, LFS induced short-term depression (*n* = 10; *p* < 0.05 vs. WT1 animals, Fig. 3A,B and Supplementary Fig. 5B). LFS did not change the expression levels of PLPP/CIN, cofilin, LIMK1, GluA1, CaMKII and CN among all experimental groups (Fig. 3C,D and Supplementary Fig. 7). LFS decreased F-actin, pCofilin, pLIMK1, pGluA1, pCaMKII and pCN levels in WT1 and Tg mice (*n* = 10, respectively; *P* < 0.05 vs. control level, Fig. 3C–E). Only pCofilin level in Tg mice was significantly lower than that in WT1 animals (*P* < 0.05 vs. WT1 animals, Fig. 3C–E). LFS also declined F-actin, pCofilin, pLIMK1, pGluA1, pCaMKII and pCN levels in WT2 mice (*P* < 0.05 vs. control level), but not KO mice (*n* = 10, respectively; Fig. 3C–E). F-actin, pCofilin, pGluA1, pCaMKII and pCN levels showed statistical difference between in WT2 and KO mice (*P* < 0.05). These findings indicate that PLPP/CIN deletion may cause dysregulation of LTD induction. Together with the data on LTP, our findings suggest that PLPP/CIN may be involved in bidirectional synaptic plasticity.

### PLPP/CIN expression affects spatial memory

**Figure 4:**
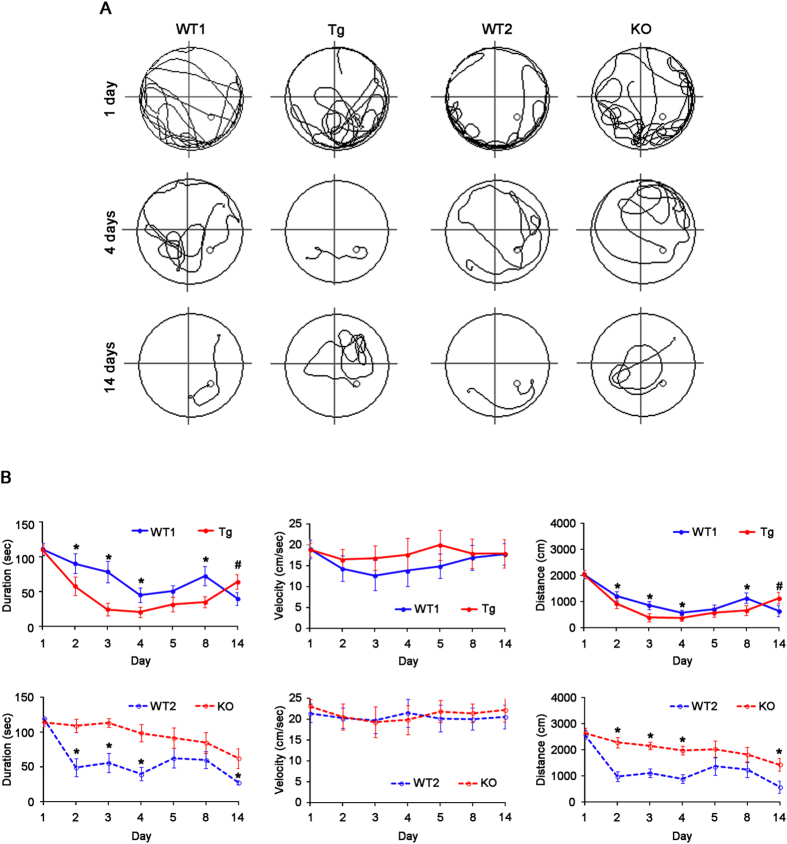
Profiles of behavioral test by Morris water maze. (**A**) Representative traces of swimming plot in Morris water maze test. (**B**) The quantitative analyses of the Morris water maze. As compared to WT animals, Tg mice rapidly improve spatial learning, while KO mice retard it over 5 days of training (*p < 0.05 vs. WT animals, n = 10, respectively). One week after the last training (14 days), Tg mice show increase in escape duration and distance (^#^*p* < 0.05 vs. 5 days, n = 10, respectively). Error bars in graphs indicates SEM.

Since dendritic spines play an important role in the hippocampus-dependent spatial memory^26^,^27^, we employed the Morris water maze test (n = 10, respectively)^28^. Over 5 days of training, all animals improved their ability to find the submerged platform, which exhibited decreasing escape duration (latency) and escape distance (total distance travelled). No difference in average swimming velocity was observed among all experimental groups (Fig. 4A,B). There was no difference in improvement of escape duration and distance with successive trials between WT1 and WT2 animals (Fig. 4B). As compared to WT animals, Tg mice showed the most rapid improvement, while KO mice exhibited retardation of spatial learning (*p* < 0.05 vs. WT animals, Fig. 4A,B). One week after the last training (at 14 days), however, Tg mice showed increase in escape duration and distance (*p* < 0.05 vs. WT1 animals, Fig. 4A,B). Together with electrophysiological data, these findings indicate that PLPP/CIN may play an important role in acquisition, consolidation and retention of memory.

### PLPP/CIN does not involve GluN trafficking, heterotrimerization and its binding with α-actinin-2

It is well known that GluN plays a pivotal role in LTP and LTD induction^5^,^29^,^30^. Since GluN is the coassembly of receptor subunit families i.e., GluN1, GluN2 and GluN3A subfamily^31^, we assessed whether PLPP/CIN associates with the dynamics of GluN distribution and affects the GluN heterotrimerization. Purification of the PSD fraction followed by immunoblotting showed that there was no difference in the GluN subunit levels in PSD fraction among four groups (Supplementary Fig. 8). Co-immunoprecipitation with GluN2A also showed that PLPP/CIN could not alter the binding of GluN2A with GluN1 or GluN2B. These findings indicate that PLPP/CIN may not affect the dynamics of GluN distribution and the heterotrimerization.

Interaction of GluN1 with α-actinin-2 (an actin binding protein) inhibits the GluN inactivation, which competes with calmodulin^32^,^33^. Therefore, we also investigated whether PLPP/CIN inhibits the interaction of GluN1 with α-actinin-2. However, there was no group wise difference in GluN1 co-precipitation with α-actinin-2 at control level (Supplementary Fig. 9). HFS increased the GluN1 association with α-actinin-2 in KO mice alone, while LFS decreased the association in WT and Tg mice (p < 0.05 vs. control level, Supplementary Fig. 9). These findings indicate that under basal condition PLPP/CIN may not affect the interaction of α-actinin-2 with GluN1; however it may be involved in GluN1 association with α-actinin-2 during synaptic plasticity.

### PLPP/CIN expression modulates the binding of regulatory molecules to GluN2A

**Figure 5:**
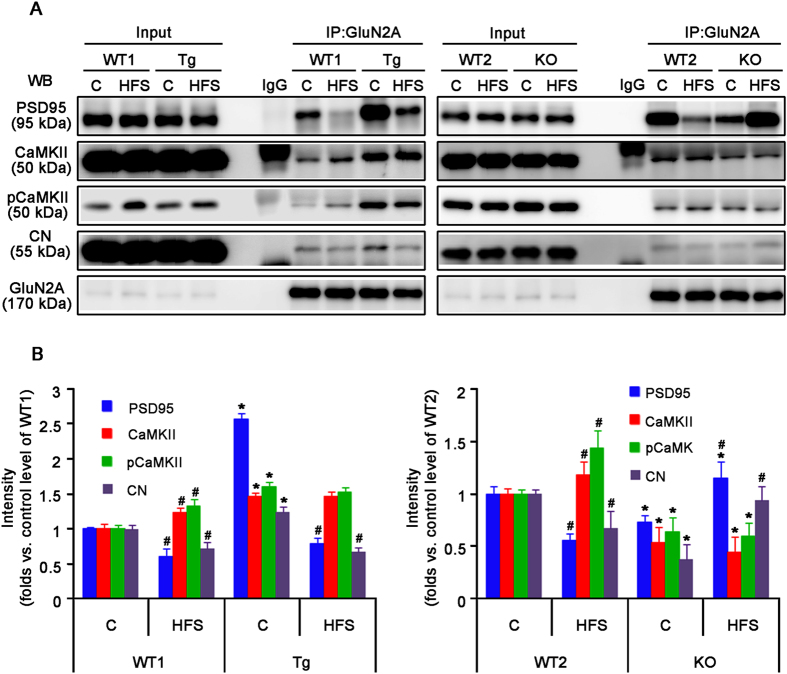
GluN2A interaction with PLPP/CIN, GluN1, PSD95, CaMKII, pCaMKII and CN in the hippocampus following LTP induction. (**A**) Co-immunoprecipitation analyses of GluN2A interaction with PSD-related postsynaptic proteins. (**B**) The quantitative analyses of co-immunoprecipitation. Under basal condition, Tg mice show significantly increase of GluN2A co-precipitation with PSD95, CaMKII and CN, whereas KO mice exhibit the reduction in GluN2A binding with these interacting proteins. HFS increases GluN2A association with CaMKII and concomitantly decreases its associations with PSD95 and CN in WT1 and WT2 mice. In Tg mice, HFS decreased the GluN2A co-precipitation with PSD95 and CN, but does not affect its association with CaMKII. In KO mice, HFS increases GluN2A co-precipitation with PSD95 and CN, but not with CaMKII (*p < 0.05 vs. WT animals, n = 10, respectively; ^#^*p* < 0.05 vs. control level; n = 10, respectively). Error bars in graphs indicates SEM.

**Figure 6:**
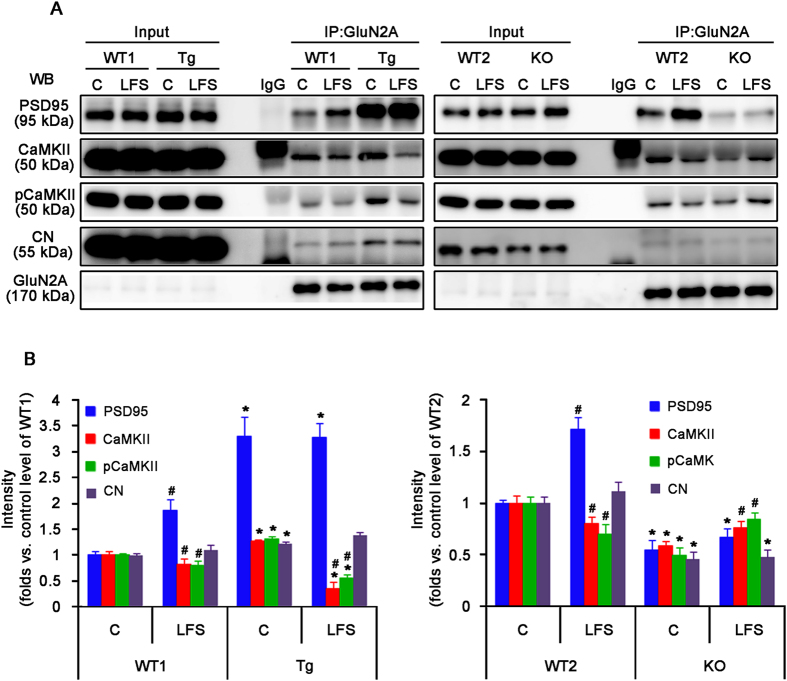
GluN2A interaction with PLPP/CIN, GluN1, PSD95, CaMKII, pCaMK and CN in the hippocampus following LTD induction. (**A**) Co-immunoprecipitation analyses of GluN2A interaction with PSD-related postysynaptic proteins. (**B**) The quantitative analyses of co-immunoprecipitation. LFS increases GluN2A association with PSD95 and concomitantly decreases its association with CaMKII in WT1 and WT2 mice. In Tg mice, LFS reduces GluN2A co-precipitation with CaMKII. In KO mice, LFS increases GluN2A association with CaMKII (*p < 0.05 vs. WT animals, n = 10, respectively; ^#^*p* < 0.05 vs. control level; n = 10, respectively). Error bars in graphs indicates SEM.

Among the GluN subunits, GluN2A plays a major role in synaptic plasticity in the adult brain^34^. GluN2A directly interacts with PSD95^35^, which is important in signal pathways controlling bidirectional synaptic plasticity^36^,^37^. Therefore, it is likely that PLPP/CIN affects GluN function by regulating the binding of regulatory molecules to GluN2A. In the present study, PLPP/CIN did not bind to GluN2A, PSD95, CaMKII and CN (Supplementary Fig. 10). Under basal condition, however, Tg mice showed significantly increase of GluN2A co-precipitation with PSD95, CaMKII and CN, while KO mice exhibited a reduction in GluN2A binding with interacting proteins. HFS increased GluN2A association with CaMKII and concomitantly decreased its associations with PSD95 and CN in WT1 and WT2 mice (*n* = 10, respectively, *p* < 0.05 vs. control level, Fig. 5). In Tg mice, HFS decreased the GluN2A co-precipitation with PSD95 and CN, but did not affect its association with CaMKII (*n* = 10, respectively, *p* < 0.05 vs. control level, Fig. 5). In KO mice, HFS increased GluN2A co-precipitation with PSD95 and CN (*n* = 10, respectively, *p* < 0.05 vs. control level, Fig. 5), but not with CaMKII. LFS increased the amount of GluN2A association with PSD95 and concomitantly decreased its association with CaMKII in WT1 and WT2 mice (*n* = 10, respectively; *p* < 0.05 vs. control level, Fig. 6). LFS reduced GluN2A co-precipitation with CaMKII in Tg mice (*n* = 10, respectively; *p* < 0.05 vs. control level, Fig. 6); whereas LFS increased GluN2A association with CaMKII in KO mice (*n* = 10, respectively; *p* < 0.05 vs. control level, Fig. 6). Unlike GluN2A, the binding of GluN2B with CaMKII, but not PSD95, was altered by HFS or LFS in all experimental groups (*n* = 10, respectively, Supplementary Fig. 11). The pattern of changed GluN2B association with CaMKII was similar to that of GluN2A association with CaMKII (*n* = 10, respectively, Supplementary Fig. 11). Therefore, our findings indicate that PLPP/CIN may regulate interactions of GluN2A with interacting postsynaptic proteins rather than GluN1 or GluN2B.

### PLPP/CIN overexpression increases neuronal activity in response to NMDA

**Figure 7:**
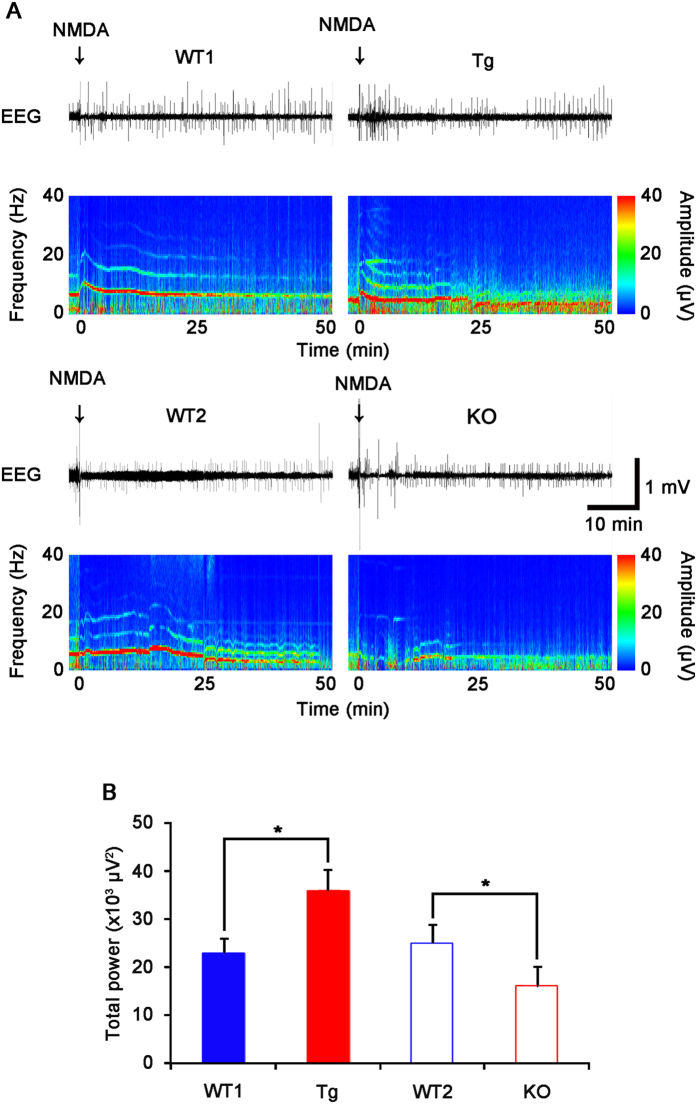
Neuronal discharge in response to NMDA (20 μM) injection into the dentate gyrus. (**A**) Representative EEG traces and frequency-power spectral temporal maps in response to NMDA injection. Arrows indicate NMDA injection time point. (**B**) Total EEG power after NMDA injection. Tg mice show increased NMDA-mediated neuronal discharge, as compared to WT1 mice. However, KO show the decline in NMDA-mediated neuronal discharge, as compared to WT2 mice (*p < 0.05 vs. WT animals; n = 5, respectively). Error bars in graphs indicates SD.

To confirm PLPP/CIN-mediated regulation of GluN activity, we applied focal NMDA injection (20 μM) into the dentate gyrus. NMDA increased the amplitude and frequency of neuronal discharges in both WT animals (*n* = 5, respectively, Fig. 7). There was no difference in the effect of NMDA on neuronal activity between WT1 and WT2 mice. The efficacy of NMDA-mediated neuronal discharge was higher in Tg mice, as compared to WT1 mice, whereas it was lower in KO mice than WT2 mice (*n* = 5, respectively; *P* < 0.05 vs. WT animals, Fig. 7). These findings indicate that PLPP/CIN may regulate increase GluN functionality via facilitating interactions of GluN2A with regulatory molecules.

## Discussion

Since the volume of a spine head is proportional to its synaptic area, the number of postsynaptic receptors, and the number of presynaptic docked vesicles^38^, many investigators postulated that persistent changes in spine shape reflects synaptic strength. However, this concept is challenged by several lines of research. LIMK1 KO mice show normal basal transmission with small dendritic spines^16^. In contrast, latrunculin B, an actin polymerization inhibitor, reduces the synaptic response without changed spine morphology^11^. In the present study, PLPP/CIN regulated the spine size, F-actin content and pCofilin level. However, PLPP/CIN overexpression or deletion did not change GABAergic neurotransmission and a short-term presynaptic plasticity (paired-pulse facilitation). These findings indicate that PLPP/CIN may not affect basal neurotransmission, and spine morphology may not represent the synaptic strength under basal condition. Furthermore, it is likely that changed spine morphology induced by PLPP/CIN is a postsynaptic event, since paired-pulse facilitation is regarded as a presynaptic phenomenon^19^.

Actin remodeling is important for the structural modification of spines by formation or removal of synapses^39^. Therefore, it is likely that larger dendritic spines have a higher possibility to enhance synaptic plasticity. However, according to the sliding threshold theory^6^, large spines prefer input to elicit LTD (inducing spine shrinkage), and small spines favor LTP induction (inducing spine enlargement). This is the possible feedback mechanism that allows spines to maintain their sizes suitable for normal synaptic strength. Indeed, LIMK1 KO mice show enhancement of LTP and normal LTD in small spines^16^. The present data reveal that Tg mice showed the enhancements of LTP and LTD with small spines, while KO mice demonstrated the disruption of both LTP and LTD with large spines. Furthermore, molecular events induced by HFS and LFS were disrupted in KO mice. Taken together, our findings indicate that PLPP/CIN may maintain appropriate sizes of dendritic spines by altered efficacy of synaptic plasticity, which would be required to limit the extent of LTP and LTD. This would be important to maintain synaptic strength and plasticity threshold in the mature brain.

F-actin at the spine neck creates a physical obstruction to diffusion^16^,^40^,^41^. The narrow neck prevents Ca^2+^ in a spine from rapidly dissipating into the dendritic shaft^42^,^43^. Therefore, spines with a thin neck are more efficient in the compartmentalization of Ca^2+^ from the dendritic shaft, as compared to spines with a thick neck^44^. In the present study, PLPP/CIN Tg mice showed thin spine necks with small spine heads. Therefore, our findings suggest that PLPP/CIN may not involve the neck constriction, but may play a distinct role in the structural maintenance of spine head. Indeed, F-actin is unnecessary for the structural support of spine necks^45^. Regardless of the role of PLPP/CIN in neck constrictions, it is simply interpreted that thin neck may involve enhanced synaptic plasticity in PLPP/CIN Tg mice. However, LIMK1 KO mice show enhanced LTP without neck constrictions^16^. In addition, synaptopodin KO mice reduces LTP with normal spine morphology^46^. Latrunculin B also inhibits both LTP and LTD without alteration in spine morphology^11^. Therefore, our findings indicate that abnormal spine morphology may reflect aberrant actin dynamics, but not the regulation of efficacy of synaptic plasticity.

The functional state of GluN is mechanosensitive and regulated by the actin cytoskeleton in Ca^2+^-dependent manner^14^,^32^,^47^. This is because F-actin depolymerization reduces GluN activity^48^, which is attributable to competitive displacement of α-actinin-2 with calmodulin to GluN1^33^,^49^. Our results indicate that no group wise difference in α-actinin-2 co-precipitation with GluN1 was observed at the control level. These findings indicate that PLPP/CIN may not involve GluN1 association with α-actinin-2 under basal condition. Interestingly, LFS decreased GluN1 co-precipitation with α-actinin-2 in WT and Tg mice, while HFS increased GluN1 co-precipitation with α-actinin-2 only in KO mice. These findings indicate that PLPP/CIN may regulate interaction of α-actinin-2 with GluN1 by activity-dependent F-actin depolymerization, which is a compensatory response to regulate appropriate GluN activity during synaptic plasticity.

**Figure 8:**
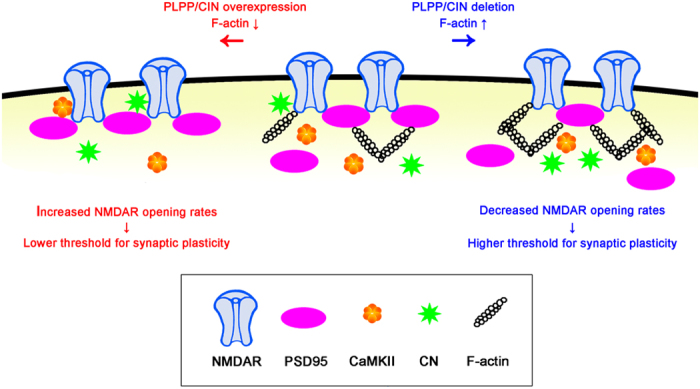
Hypothesized roles of the PLPP/CIN in GluN2A interaction with postsynaptic proteins for regulating the plasticity threshold.

GluN2A association with interaction proteins plays an important role in synaptic plasticity^35^. Interestingly, the present data reveal that PLPP/CIN regulated GluN2A co-precipitation with PSD95, CN and CaMKII under basal condition, although PLPP/CIN did not bind with GluN2A PSD95, CaMKII and CN. Furthermore, HFS and LFS changed GluN2A association with interacting proteins, accompanied by altered F-actin level. Since F-actin acts as an anchor for PSD scaffolding proteins^49^,^50^, it is presumable that PLPP/CIN may regulate the interaction of GluN2A and regulatory proteins by local F-actin depolymerization, which in turn could affect synaptic efficacy. In other words, F-actin lattice in spine may hinder the binding of GluN2A with PSD-related regulatory molecules. Indeed, F-actin depolymerization redistributes postsynaptic proteins^15^ and gates protein translocations into spines^9^. Renner *et al*.^51^ also reported the presence of actin-mediated obstacles and barriers for the flux of synaptic molecules, which influence synaptic activity. Indeed, the rapid and reversible motility of actin polymers represents a critical window of opportunity allowing protein translocation to occur prior to longer-term stabilization of spines^9^. Therefore, PLPP/CIN may provide an opportunity for rapid motility of PSD-related molecules by eliminating the local F-actin barrier that enables the bindings of GluN2A with regulatory proteins during dendritic spine reorganization (Fig. 8).

Although PSD95 does not govern synaptic GluN currents, subunit expression, localization and synaptic morphology^52^, it plays a critical role in regulating the gating, trafficking and intracellular signal pathways of intact GluN control of bidirectional synaptic plasticity^36^,^37^,^53^,^54^. PSD95 reportedly affects efficacies of LTP and LTD^52^,^55^,^56^. Indeed, Gardoni *et al*.^57^ reported that LTP induction entails dissociation of PSD95 from GluN. In the present study, neither HFS nor LFS affected GluN2B co-precipitation with PSD95 in all experimental groups. However, HFS decreased GluN2A association with PSD95 in WT and Tg mice, whereas HFS increased the association in KO mice. In contrast, LFS increased GluN2A co-precipitation with PSD95 in both WT mice, but not in Tg and KO mice. These findings indicate that in Tg mice the saturation of GluN2A association with PSD95 under basal condition may limit additional binding of PSD95 to GluN2A during LTD. Furthermore, in KO mice reduction in GluN functionality under basal condition may alter the efficacy of synaptic plasticity in response to HFS to LTD-like event. These findings suggest that PLPP/CIN may shift the threshold of synaptic plasticity by modulating the activity-dependent shuttling of PSD95 to and from the GluN2A. This hypothesis is also supported by the changed efficacy of NMDA-mediated neuronal discharges in KO and Tg mice.

Whereas deficits in spatial memory is relevant to abnormal synaptic plasticity, PSD95 and LIMK1 KO mice show an inverse relationship between LTP and hippocampus-dependent learning^16^,^52^. In the present study, Tg mice showed an increase in the acquisition of spatial memory as compared to WT1 animals. However, Tg mice showed increase in escape duration and distance at 1 week after the last training (at 14 days), which indicates impaired retention of memory. In contrast, KO mice exhibited the retardation of spatial learning. These findings are consistent with our eletrophysiological data, and suggest that PLPP/CIN may be involved in memory formation by rapidly encoding new information and easily erasing it.

In the present study, LFS decreased both pGluA1-S831 and -S845 in WT1, Tg and WT2 mice, but not KO mice. NMDA-induced LTD-like synaptic depression induces a dramatic dephosphorylation of pGluA-S845, with less dephosphorylation of pGluA1-S831 in mouse^20^. Unlike chemical LTD, electrical LTD produces specific dephosphorylation of pGluA-S845, but not pGluA1-S831^21^. However, LTD induction in naïve synapses results in the dephosphorylation of pGluA-S845, while LTD induction in previously potentiated synapses leads to the dephosphorylation of pGluA1-S831^21^. In addition, Ho *et al*.^22^ reported that LFS also decreases pGluA1-S831 level accompanied by reduction in pCaMKII level. Furthermore, CN opposes protein kinase A phosphorylation of pGluA1-S845 to restrict synaptic incorporation of Ca^2+^-permeable GluA1^23^. Thus, it is likely that LFS may decrease both pGluA1-S831 and -S845 by reduced CaMKII activity and increased CN activity, respectively, and that in KO mice disrupted molecular events induced by HFS and LFS may be consequences from the low efficacy of synaptic plasticity.

On the other hand, our previous study demonstrated that Tat-PLPP/CIN transduction increases the excitability ratio and population spike amplitude ratio in response to 20 ms interstimulus interval^17^. Furthermore, HSF decreases PLPP/CIN protein expression level in the rat dentate gyrus^10^. However, we could not find these alterations in the present study. These discrepancies could have resulted from differential methodology. Firstly, the distinct effect of genetic approaches and Tat-PLPP/CIN on properties of spines may evoke these discrepancies. The increase in population spike amplitude ratio induced by Tat-PLPP/CIN recovers to the basal level 1.5 h after transduction^17^. Therefore, it is likely that Tat-PLPP/CIN may evoke the transient imbalance in spine homeostasis. Indeed, Tat-PLPP/CIN transduction transiently decreases the efficiency of LTP induction in the rat dentate gyrus without changes in drebrin (a spine marker) positive structures^10^. Unlike transient exogenous PLPP/CIN transduction^17^, the present study reveals that PLPP/CIN overexpression and its deletion changed the morphological properties of spines. Thus, genetic approaches of PLPP/CIN may activate long-lasting adaptive postsynaptic events in response to alteration in morphological properties of spines, which was observed in efficacy of synaptic plasticity in the present study. Similar to the present study, genetic inhibition of actin polymerization by LIMK1 deletion shows normal basal transmission with small dendritic spines^16^, while transient chemical inhibition of actin polymerization by latrunculin B reduces the synaptic response without changed spine morphology^11^. Secondly, the differential methodology of LTP induction between a previous and the present study would make distinct responses of PLPP/CIN expression to HFS. In a previous study^10^, PLPP/CIN protein expression level is markedly decreased in rat dentate gyrus following total 2000 HSF. In the present study, however, we could not find the reduction in PLPP/CIN expression level induced by total 900 HFS. Since PLPP/CIN activity was dependent to HFS intensity^10^, it is likely that differential stimulus intensity and frequency methodologies of LTP induction would result in these discrepancies.

In summary, we provided novel evidence that PLPP/CIN is intimately involved in controlling dendritic spine as well as synaptic plasticity. The present study also proposes for the first time the role of PLPP/CIN in GluN functionality via regulation of GluN2A interaction with postsynaptic proteins (particularly PSD95), which profoundly shifts the threshold balance between LTP and LTD (Fig. 8). Therefore, our findings suggest that PLPP/CIN may play an important role in information storage and recall capacity, which manifests as a learning memory.

## Methods

Extended Experimental Procedures can be found in Supplemental Information.

### Experimental animals and chemicals

Tg mice and KO mice were generated from Macrogen (Seoul, South Korea) and Taconic biosciences, Inc (NY, USA), respectively. Animals were provided with a commercial diet and water *ad libitum* under controlled temperature, humidity and lighting conditions (22 ± 2 °C, 55 ± 5% and a 12:12 light/dark cycle). All reagents were obtained from Sigma-Aldrich (St. Louis, MO, USA), except as noted. The procedures involving animals and their care were conducted in accord with our institutional guidelines that comply with NIH Guide for the Care and Use of Laboratory Animals (NIH Publications No. 80-23, 1996). We have made all efforts to minimize the number of animals used and their suffering. All experimental protocols were approved by the Animal Care and Use Committee of Hallym University.

### Immunohistochemistry

Animals were anesthetized with urethane anesthesia (1.5 g/kg, I.P.) and perfused transcardially with 4% paraformaldehyde in 0.1 M phosphate buffer (PB, pH 7.4). Brains were post-fixed in the same fixative overnight and then cryoprotected and sectioned at 30 μm with a cryostat. Free-floating coronal sections were incubated in PLPP/CIN antibody in PBS containing 0.3% Triton X-100 overnight at room temperature. Tissue sections were developed in 3,3′-diaminobenzidine in 0.1 M Tris buffer and mounted on gelatin-coated slides. For double immunofluorescent study, sections were incubated in a mixture of PLPP/CIN and GFAP antibody in PBS containing 0.3% Triton X-100 and 2% normal chicken serum overnight at room temperature. Sections were also incubated in a mixture of FITC- and Cy3-conjugated secondary antisera (Amersham, Piscataway, NJ, USA, 1:200) for 1 h at room temperature. Sections were mounted in Vectashield mounting media with DAPI (Vector). Immunoreaction was observed using an Axio Scope microscope (Carl Zeiss). To establish the specificity of the immunostaining, a negative control test was carried out with preimmune serum instead of the primary antibody. No immunoreactivity was observed for the negative control in any structures.

### Golgi impregnation and analysis of spine morphology

Golgi impregnation was performed using FD Rapid GolgiStain™ kit (FD NeuroTechnologies, Inc., MD, USA), according to the manufacturer’s instructions. Thereafter, dendritic spine and dendritic tree morphology were analyzed using an AxioImage M2 microscope and AxioVision Rel. 4.8 software. Spine density was calculated as the total dendritic spine count per total dendritic length. Spine head diameters were measured at individual *z* sections where the spine is in focus by using AxioVision Rel. 4.8 software. The categories of spine classification was performed according to traditional guidelines as followed; (1) Filopodia-like spines were relatively long and thin and did not exhibit a bulbous head. (2) Stubby spines did not have a clear neck, and showed the diameter of the head was similar to the total length of the spine. (3) Thin spines showed neck length much larger than its diameter, and diameter of the head larger than diameter of the neck. (4) Mushroom spines showed the diameter of the head was much larger than diameter of the neck.

### Electrophysiology

The procedures for electrophysiological recordings were described previously^10^,^58^. Briefly, animals were anesthetized (urethane, 1.5 g/kg, I.P.) and placed in a stereotaxic frame. Rectal temperature was maintained at 36.5 ± 0.5 °C using a homoeothermic temperature control unit. The skull was exposed and two small holes were drilled over the dentate gyrus (2 mm posterior; 1.25 mm lateral, 2–2.5 mm depth from bregma) and the angular bundle (3.8 mm posterior; 2 mm lateral; 2.5–3 mm depth from bregma). A monopolar recording electrode was positioned in the dentate gyrus, and a bipolar stimulating electrode was positioned in the angular bundle. Electrode depths were set by optimizing the evoked responses. The reference electrode was placed in the posterior cranium over the cerebellum. For measurement of paired-pulse response, stimuli were delivered at interstimulus intervals of 20, 30, 70, 150 and 250 ms as DC square pulses at 0.1 Hz with pairs of 100 μs constant current stimuli, after establishing a stable baseline for at least 30 min and a control input-output (IO) curve. Synaptic responses were monitored by applying single stimuli every 1 min at an intensity sufficient to elicit 50% maximal population spike amplitudes. For LTP and LTD induction, the stimulus intensity that produced a half maximal amplitude population spike was applied. LTP was induced by 900 total stimuli, delivered in three 1 s long tetanic (300 Hz) stimulus trains, 1 min apart. LTD was induced by 900 total stimuli, delivered in continuous 1 Hz stimulus trains. Responses were recorded at 10 min intervals after tetanus for 2 h. Signals were recorded with DAM 80 differential amplifier (0.1–3,000 Hz bandpass, World Precision Instruments, USA) and data were digitized (20 kHz) and analyzed on LabChart Pro v7 (AD Instruments, Australia). To analyze changes in evoked response, all of the population spike amplitude and fEPSP slope measurements during recording session were normalized by the averages of the population spike amplitude and fEPSP slope during the baseline measurement. After recording, the animal was quickly decapitated for Western blot and co-immunoprecipitation. For analysis of GluN functionality, animals were injected with saline or pilocarpine into the hippocampus, after baseline recording for at least 30 minutes. EEG signals were digitized and analyzed using LabChart Pro v7 (AD Instruments, NSW, Australia). Spectrograms were automatically calculated using a Hanning sliding window with 50% overlap.

### Analysis of F-actin content and GluN localization in PSD fraction

To analyze F-actin content, we used G-actin/F-actin *in vivo* assay biochem kit (Cytoskeleton, Inc, USA) and subcellular Protein Fractionation Kit for Tissues (Thermo scientific, USA), respectively, according to the manufacturer’s instructions. Preparation of crude PSD fractions was performed according to previously published procedures^59^ with some modifications. Hippocampal tissues were homogenized in the homogenate buffer (0.32 M sucrose, 50 mM Tris-HCl pH 7.4, 0.1 mM PMSF, 1 mM EGTA, plus protease inhibitors (Roche)) and centrifuged at 1000 G for 20 min. The supernatants were then centrifuged at 10000 G for 15 min. The pellets were resuspended with homogenate buffer and centrifuged at 25,000 G for 20 min. The pellets were resuspended with homogenate buffer and further separated on a sucrose density gradient (0.8 and 1.5 M sucrose) by ultracentrifugation at 90000 G for 2 h at 2 °C. To prepare PSD fraction, fractions were further solubilized with 0.5% Triton X-100 and then centrifuged at 200000 G for 1 h. The resulting pellets were considered to be the purified PSD fraction whereas the supernatants served as the non-PSD fraction. The resulting pellet (PSD fraction) was suspended in 1% SDS, 40 mM Tris-HCl, pH 8.0 and diluted in SDS sample buffer. Protein sample of each fraction was heated at 100 °C for 5 min in the presence of 2-mercaptoethanol. Next, Western blotting was performed according to standard procedures (see below).

### Immunoblotting

The right hippocampus (ipsilateral to stimulus) and the left hippocampus (contralateral to stimulus) were used as test and control tissues, respectively. After recording, the animal was quickly decapitated, and their hippocampi were dissected out in the presence of cooled artificial cerebrospinal fluid (in mM: 124 NaCl, 5 KCl, 1.25 NaH_2_PO_4_, 26 NaHCO_3_, 10 dextrose, 1.5 MgCl_2_, and 2.5 CaCl_2_). A block of dentate gyrus (~1 mm^3^) was excised from the hippocampus, and each was placed in individual, coded vials on dry ice, which then were stored at −80 °C until biochemical analysis. The microdissected tissue samples were homogenized in 50 mM Tris containing ethylene glycol tetraacetic acid (pH 8.0), 10 mM ethylenediaminetetraacetic acid (pH 8.0), 0.2% Tergitol type NP-40, 15 mM sodium pyrophosphate, 100 mM β-glycerophosphate, 2 mM sodium orthovanadate, 150 mM NaCl, 1 mM phenylmethylsulfonyl fluoride, 50 mM NaF, 50 mM HEPES (pH 7.4) and 1 mM dithiothreitol (DTT) containing protease inhibitor cocktail (complete, Roche Applied Sciences), phosphatase inhibitor cocktail (PhosSTOP^®^, Roche Applied Science). The protein concentration in the supernatant was determined using a Micro BCA Protein Assay Kit (Pierce Chemical, Rockford, IL, USA). Tissue lysate proteins were loaded into a polyacrylamide gel. After electrophoresis, gels were transferred to nitrocellulose transfer membranes (Schleicher and Schuell BioScience Inc., Keene, NH, USA). The filters were pre-blocked with 5% nonfat dry milk in tris-buffered saline (TBS) containing 0.1% Tween 20 for 45 min, followed by incubation with each primary antibody (Supplementary Table 1). Subsequently, membranes were reacted with an HRP-conjugated secondary antibody. Membrane was developed with an ECL Western Blotting Detection Kit (GE Healthcare, Piscataway, NJ, USA). The rabbit anti-β-actin primary antibody was used as internal reference. The signals were scanned and quantified on ImageQuant LAS4000 system (GE health). The values of each sample were normalized with the corresponding amount of β-actin.

### Co-immunoprecipitation

The microdissected tissue samples were lysed in radioimmune precipitation buffer (RIPA: 50 mM Tris–HCl pH 8.0; 1% Nonident P-40; 0.5% deoxycholate; 0.1% SDS) containing protease inhibitor cocktail (complete, Roche Applied Sciences), phosphatase inhibitor cocktail (PhosSTOP^®^, Roche Applied Science) and 1 mM sodium orthovanadate. Protein concentrations were determined by BCA protein assay (Pierce) and equal amounts of total proteins were precipitated with the appropriate primary antibodies and protein G sepharose at 4 °C overnight. Beads were collected by centrifugation, eluted in 2× SDS sample buffer and boiled at 95 °C for 5 min. Next, Western blotting was performed according to standard procedures (see above).

### Morris water maze

Spatial learning and memory were tested by the Morris water maze hidden platform task using the same maze and protocol as described^60^.

### Quantification and statistical analysis

Data were analyzed by one-way analysis of variance (ANOVA) coupled Tukey’s test for multiple comparison or Student *t*-test. For the behavioral studies, data were analyzed by ANOVA for differences between the genotypes. Probe trial scores within experimental groups were evaluated by paired Student *t*-test. Differences were considered as significant for p < 0.05.

## Additional Information

**How to cite this article**: Kim, J.-E. *et al*. PLPP/CIN regulates bidirectional synaptic plasticity via GluN2A interaction with postsynaptic proteins. *Sci. Rep*. **6**, 26576; doi: 10.1038/srep26576 (2016).

## Supplementary Material

Supplementary Information
