# Supplementary material for: PLPP/CIN regulates bidirectional synaptic plasticity via GluN2A interaction with postsynaptic proteins
Source: Sci Rep. 2016 May 23;6:26576. doi: 10.1038/srep26576 (PMC4876383; doi:10.1038/srep26576)
Supplement: Supplementary Information [file srep26576-s1.pdf]

Supplementary information

PLPP/CIN regulates bidirectional synaptic plasticity via GluN2A interaction with postsynaptic proteins

Ji-Eun Kim[1], Yeon-Joo Kim, [1] Duk-Shin Lee[1], Ji Yang Kim[1], Ah-Reum Ko[1],

Hye-Won Hyun[1], Min Ju Kim[1], Tae-Cheon Kang[1]

[1]Department of Anatomy and Neurobiology, Institute of Epilepsy Research, College of Medicine, Hallym University, Chuncheon 200-702, South Korea.

* Correspondence should be addressed to T-C K (e-mail: tckang@hallym.ac.kr)

Supplementary methods

Generation of human PLPP/CIN transgenic mice

A full-length untagged cDNA encoding human *PLPP/CIN* including 5'- and 3'- UTR from pQE30-hPDXP plasmid (a gift from Dr. O.S. Kwon, Kyungpook National University, Korea) was subcloned into Kpn I and ExoRI site of pEF1a promoter vector, which contains the mouse EF1a promoter and a BGH poly-A tail. The resulting plasmid, pEF1a- *PLPP/CIN*, was digested using NruI and Drd I to isolate the transgenic cassette (Supplementary Figure 1). The isolated cassette was injected into C57BL/6 mouse embryos and the mouse embryos (fertilized one-cell zygotes) were then implanted into female mice. PLPP/CIN Tg mice were identified by PCR analysis using genomic DNA prepared from their tails. Positive founder mice were identified using a forward primer (5'-TAT GCG ATG GAG TTT CCC CAC-3') and a reverse primer (5'-TGT TGC TCA CAA ACA GAG CCG-3'). PCR condition was as followed; After initial denaturation at 94 ℃ for 5 min, 35 cycles of primer annealing and elongation were performed at 56 ℃ for 1 min and 72 ℃ for 10 min. Positive founder line was identified and mated to C57BL/6J mice.

Generation of PLPP/CIN KO mice

The PLPP/CIN KO mice were generated by Taconic biosciences, Inc (USA). Supprementary Figure 2 demonstrates the PLPP/CIN targeting strategy. The target vector was electroporated into 129/SvEv ES cells. ES cell clone was microinjected into C57BL/6J (albino) blastocysts. Resulting chimeras were mated to C57BL/6J (albino) females to generate mice that were heterozygous mice. Heterozygous mice were intercrossed, yielding KO mice. PLPP/CIN KO mice were identified by PCR analysis using genomic DNA prepared from their tails. Gene specific primers pair (wild-type oligomer: TACTATGTGGAGAGCATTGCGG or mutant oligomer: ACAAACCTTCCTGGATCCCAGC) together with neomycin specific primer pair (GCAGCGCATCGCCTTCTATC) were employed. PCR was carried out at initial denaturing step of 94 ℃ for 5 min followed by 30 cycles of 94℃ (15 sec), 65℃, (30 sec), 72 ℃, (30 sec), 94℃ (15 sec), 55℃, (30 sec), then a final amplification step at 72℃ for 30 sec.

Morris water maze

The sequence of testing was performed as followed: In Day 1-5, spatial learning and memory were measured. In day 8 and 14, reversal learning (oblivion of learning) was tested. The platform stayed in the same quadrant

and the animals were released from four different positions at the pool perimeter. Mice performed four trials per a day on five consecutive days with a maximum length of 120 s and an inter-trial interval of 15 min. If mice did not find the platform, animals were gently guided to the platform. Spatial learning was assessed to measure the latency to find the platform and the swimming distance. General activity was assessed by swim speed. Learning capabilities were assessed to measure the time to find the platform (latency) and the swimming distance each mouse spent searching for the platform at probe trial. For evaluation of reversal learning (3 and 7 days after spatial learning test), four trials were performed with the former platform location. A computerized video system registered moving-path and duration in water maze tests automatically. The software used for data acquisition and analysis was EthoVision XT® release 8.0 (Noldus Information Technology, Utrecht, Netherlands).

Supplementary Figures

A

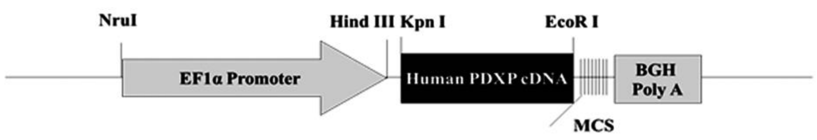

B

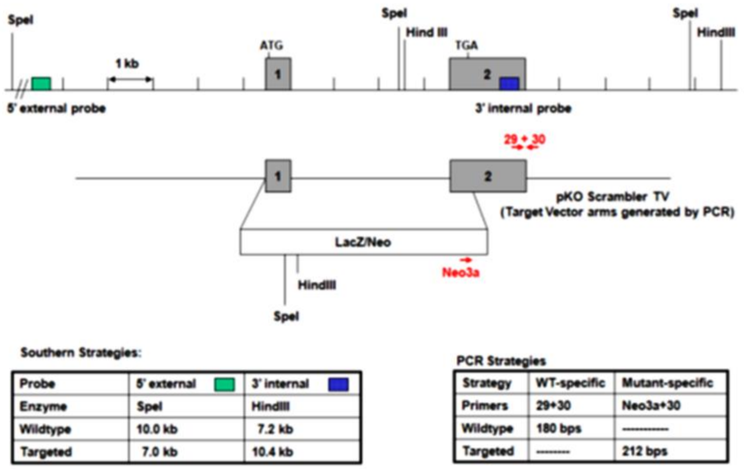

Supplementary Figure 1. Strategy of PLPP/CIN vector construction (A) and PLPP/CIN targeting strategy (B).

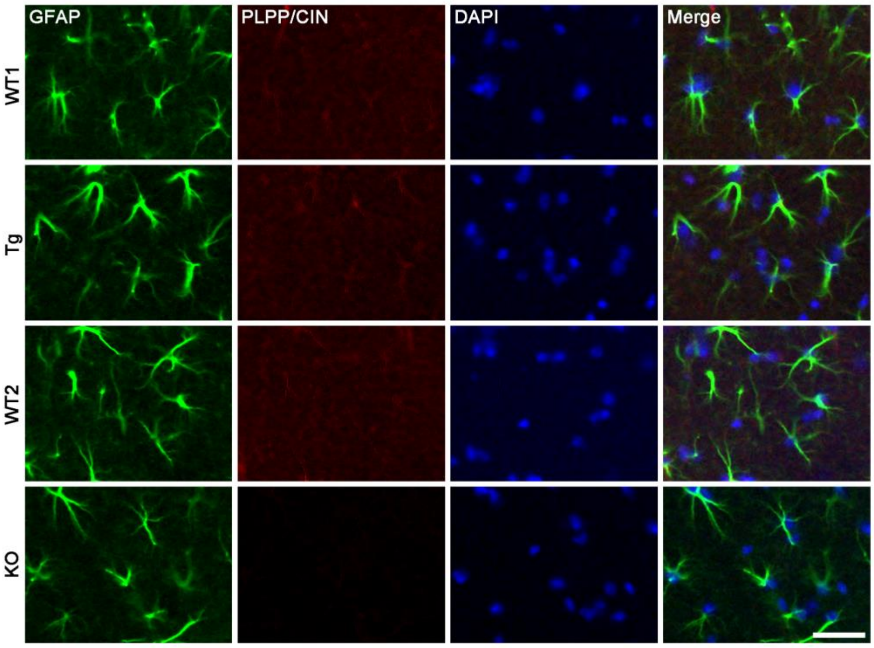

Supplementary Figure 2. Double immunofluorescent images for GFAP and PLPP/CIN. Astroglial PLPP/CIN expression level is very low, and nearly undetectable. Astroglial PLPP/CIN expression in Tg mice is similar to that in WT1 mice. Bar = 12.5 μm.

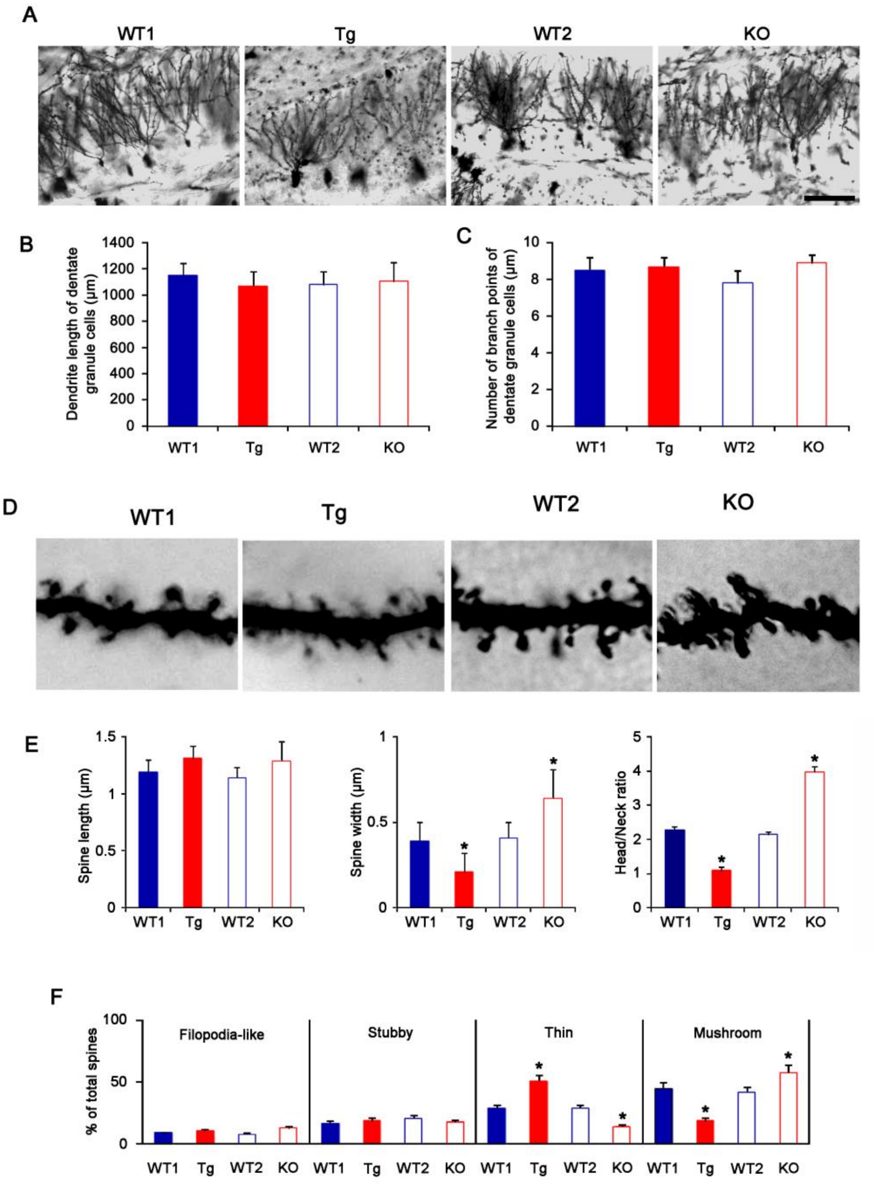

Supplementary Figure 3. The representative photos of dendritic trees in dentate granule cells and spine morphology in CA1 pyramidal cells in WT, Tg and KO mice. (A) Dendritic trees are similarly observed in all animal groups. Bar = 100 μm. (B, C) There is no difference in dendritic length and number of branch points of dentate granule cells among four group (n = 7, respectively). (D) Representative spine morphology of CA1 pyramidal cells. Bar = 5 μm. (E) Spine length, spine width and head/neck ratios of CA1 pyramidal cells. (*p < 0.05 vs. WT animals; n = 7, respectively). Error bars in graphs indicates SD. (F) Percentage of categories of spine classification in dentate granule cells. (*p < 0.05 vs. WT animals; n = 7, respectively). Error bars in graphs indicates SEM.

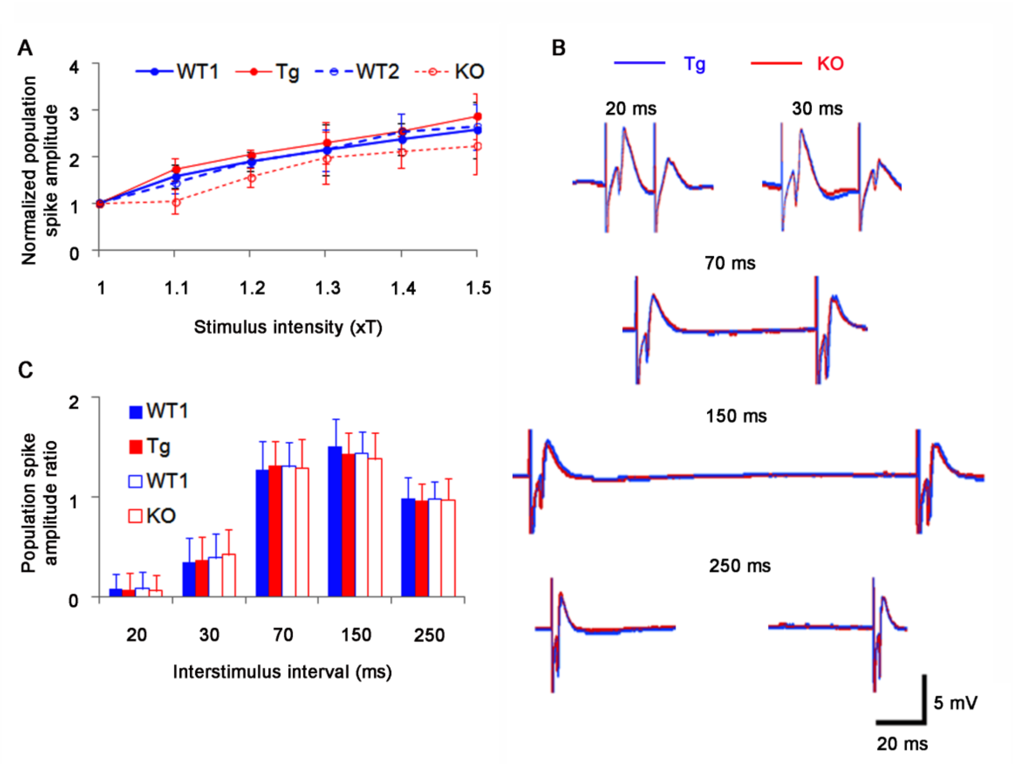

Supplementary Figure 4. Profiles of paired-pulse responses of the dentate gyrus of WT1, Tg, WT2 and KO mice. (A) Input-output (IO) curves of WT1, Tg, WT2 and KO mice. Error bars in graphs indicates SEM. (B) Representative traces of paired-pulse responses in the dentate gyrus of Tg and KO mice. (C) The normalized population spike amplitude ratio of the dentate gyrus in response to ×T 1.5 stimulus intensity. There is no difference in the normalized population spike amplitude ratio at any interstimulus interval among four groups (n = 10, respectively). Error bars in graphs indicates SEM.

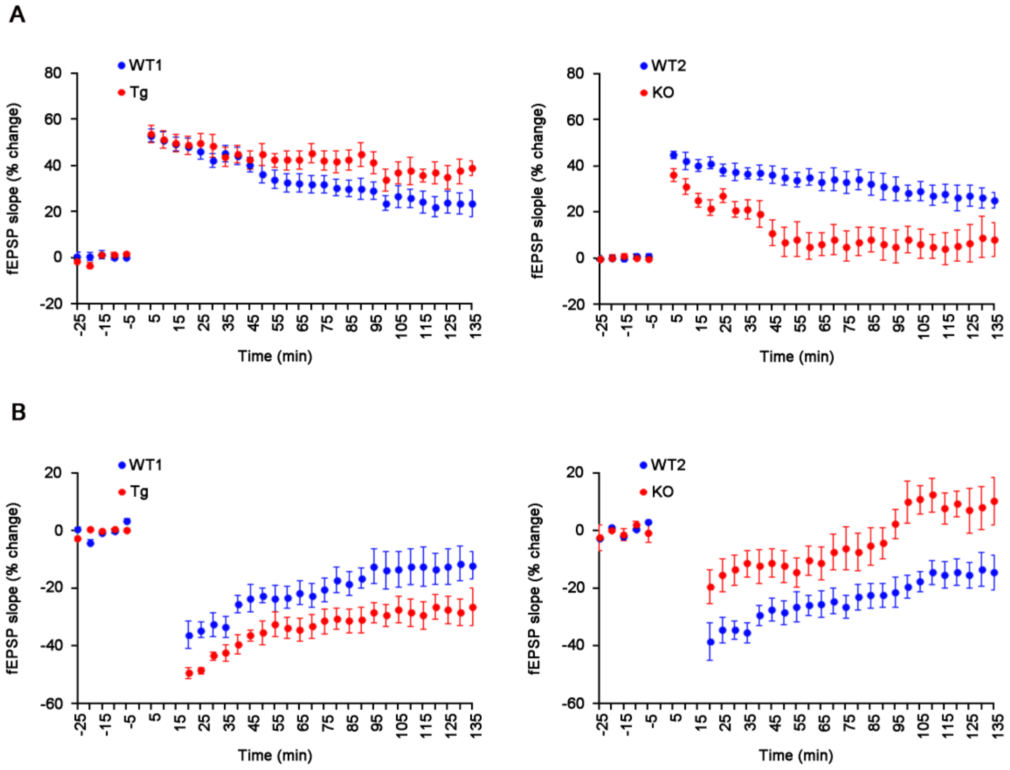

Supplementary Figure 5. Profiles of fEPSP slope induced by HFS (A) and LFS (B). (A) The quantitative analyses of fEPSP slope during LTP induction. As compared to WT animals, Tg mice show enhanced LTP induction, while KO mice exhibit disrupted LTP induction ($p < 0.05$; $n = 10$, respectively). Error bars in graphs indicates SEM. (B) The quantitative analyses of fEPSP slope during LTD induction. As compared to WT animals, Tg mice show enhanced LTD induction, while KO mice exhibit short-term depression ($p < 0.05$; $n = 10$, respectively). Error bars in graphs indicates SEM.

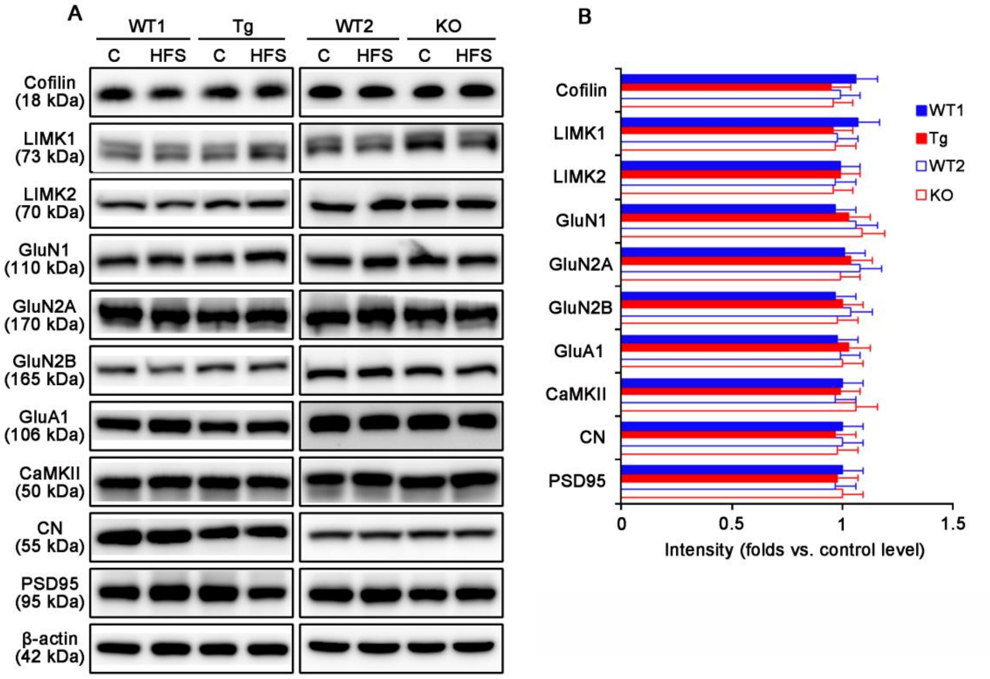

Supplementary Figure 6. Effect of HFS on protein expression in the dentate gyrus. Western blot analysis reveals that HFS cannot affect Cofilin, LIMKs, GluN subunits, GluA1, CaMKII, CN and PSD95 expression levels (n = 10, respectively). Error bars in graphs indicates SEM.

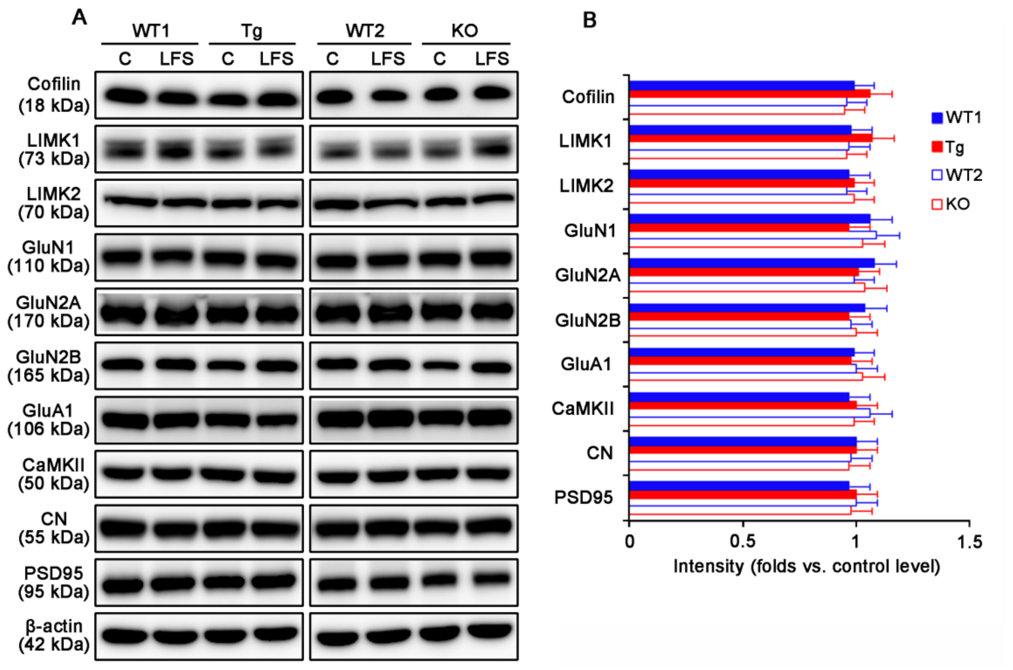

Supplementary Figure 7. Effect of LFS on protein expression in the dentate gyrus. Western blot analysis reveals that LFS cannot affect Cofilin, LIMKs, GluN subunits, GluA1, CaMKII, CN and PSD95 expression levels (n = 10, respectively). Error bars in graphs indicates SEM.

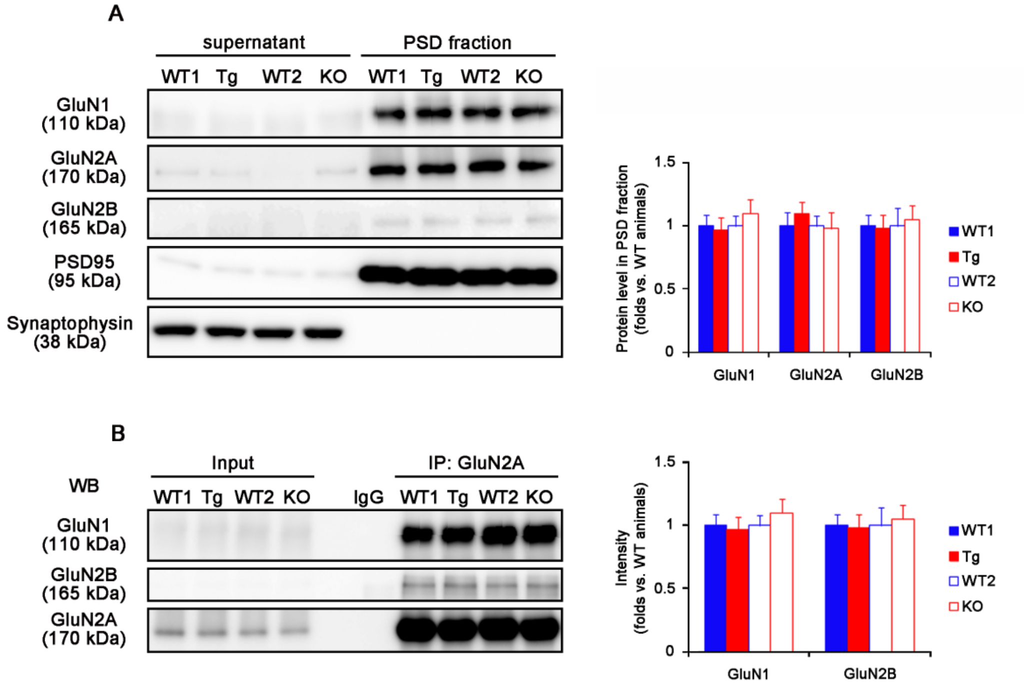

Supplementary Figure 8. The GluN distribution in PSD fraction and its GluN heterotrimerization. (A) No difference in GluN1, GluN2A and GluN2B subunit level in PSD and non-PSD (Supernatants) fractions among four groups (n = 10, respectively). Error bars in graphs indicates SEM. (B) Co-immunoprecipitation of GluN2A with GluN1 and GluN2B shows that no difference in GluN heterotrimerization among four groups (n = 10, respectively). Error bars in graphs indicates SEM.

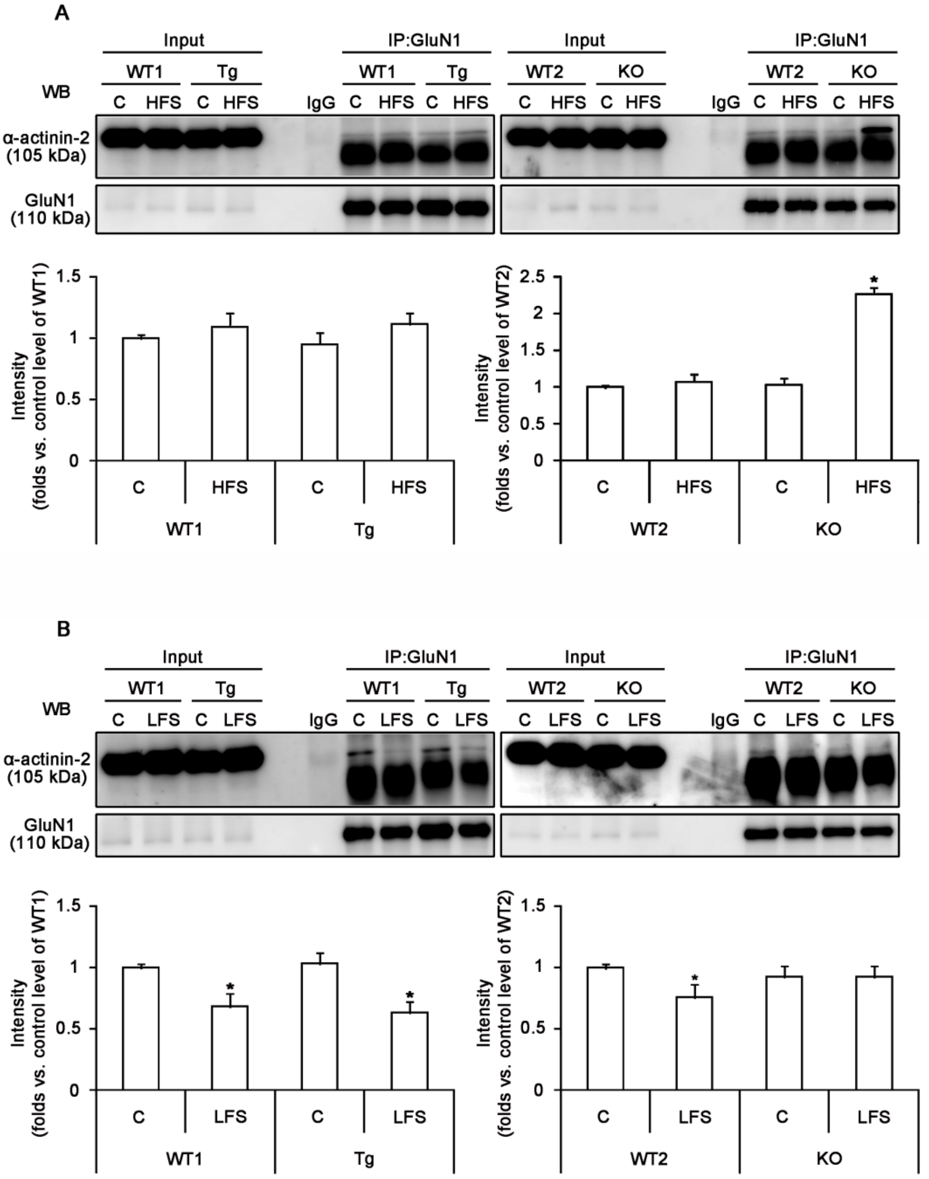

Supplementary Figure 9. The characteristics of GluN1 co-precipitation with α-actinin-2 in the hippocampus induced by LTP and LTD induction. (A) Co-immunoprecipitation analyses of GluN1 interaction with α-actinin-2 in the hippocampus induced by HFS. There is no difference in GluN1 association with α-actinin-2 under basal condition. HFS increases the GluN1 co-precipitation with α-actinin-2 only in KO mice (*$p < 0.05$ vs. control level; $n = 10$, respectively). Error bars in graphs indicates SEM. (B) Co-immunoprecipitation analyses of GluN1 interaction with α-actinin-2 in the hippocampus induced by LFS. LFS decreases GluN1 association with α-actinin-2 in WT and Tg mice, not KO mice (*$p < 0.05$ vs. control level; $n = 10$, respectively). Error bars in graphs indicates SEM.

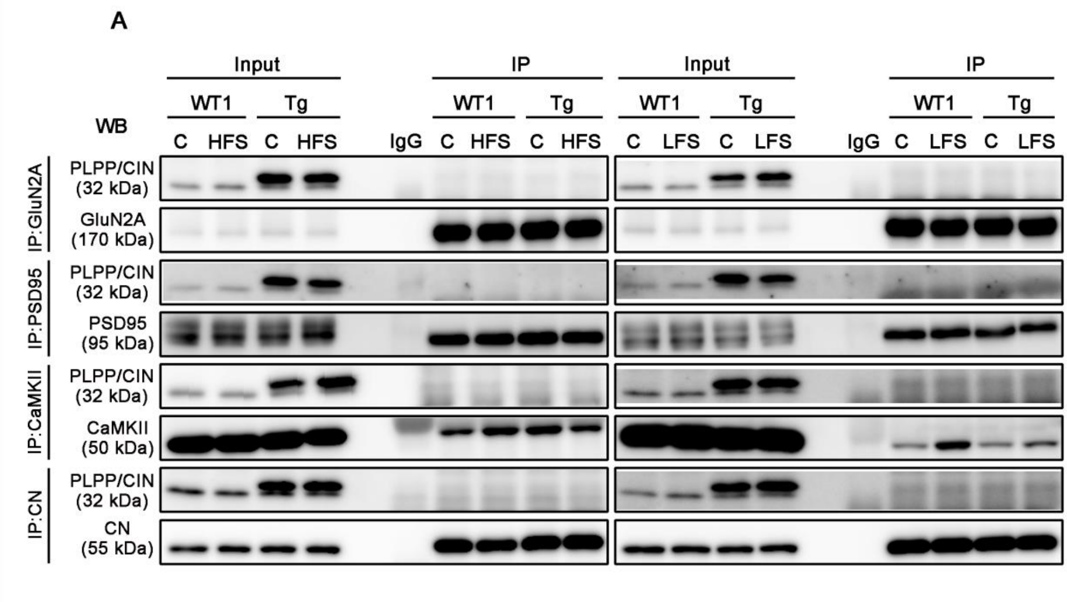

Supplementary Figure 10. The characteristics of PLPP/CIN co-precipitation with GluN1, PSD95, CaMKII and CN in the hippocampus induced by LTP and LTD induction. Co-immunoprecipitation analyses reveal that PLPP/CIN cannot interact with CaMKII, CN, GluN1, GluN2A and PSD95.

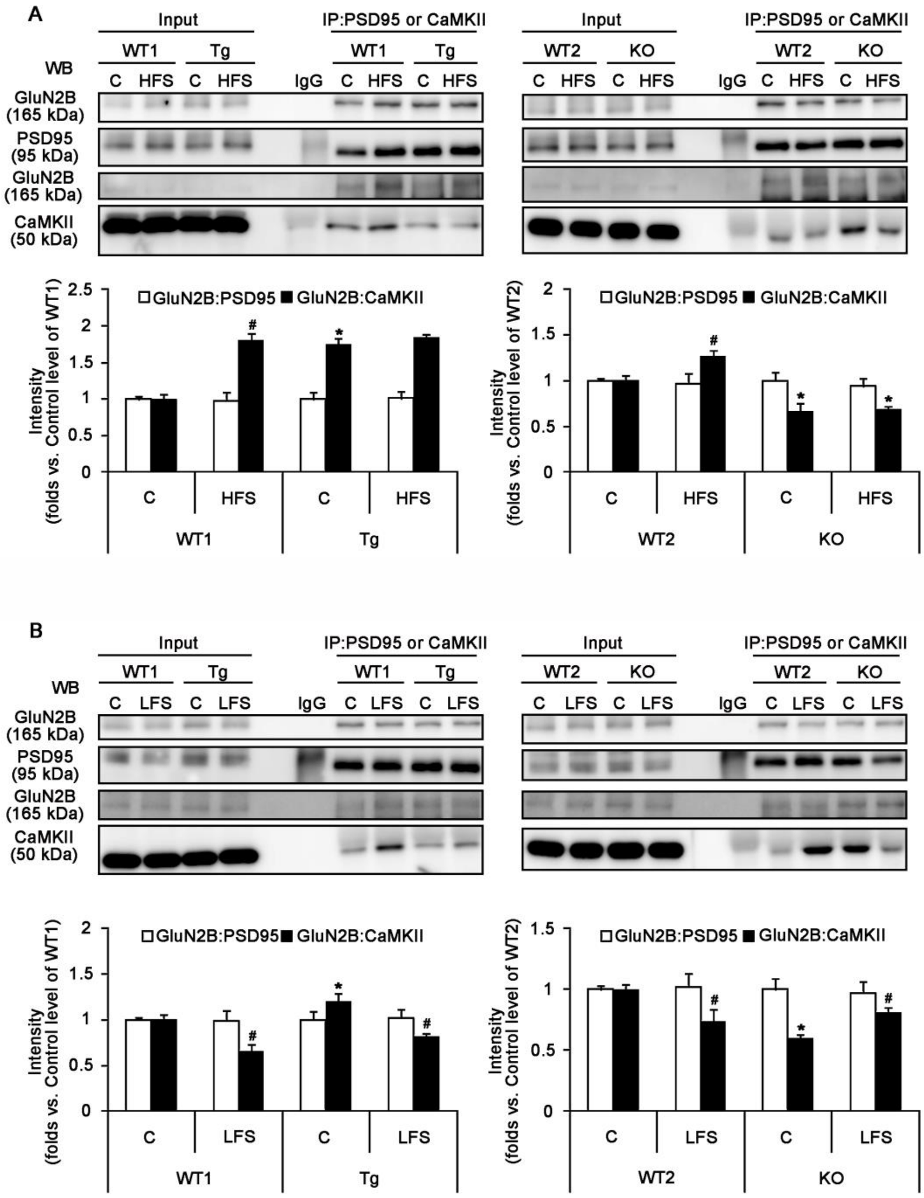

Supplementary Figure 11. The characteristics of PSD95 and CaMKII co-precipitations with GluN2B in the hippocampus induced by LTP (A) and LTD (B) induction. Co-immunoprecipitation analyses reveal that PSD95 association with GluN2B is unaltered by HFS and LFS. However, HFS increases GluN2B association with CaMKII in WT1 and WT2 animals. In Tg and KO mice, HFS does not affect its association with CaMKII. In addition, LFS decreases GluN2B association with CaMKII in WT1 and WT2 mice. In Tg mice, LFS reduces GluN2B co-precipitation with CaMKII. In KO mice, LFS increases GluN2B association with CaMKII (*$p < 0.05$ vs. WT animals, #$p < 0.05$ vs. control level; n = 10, respectively). Error bars in graphs indicates SEM.

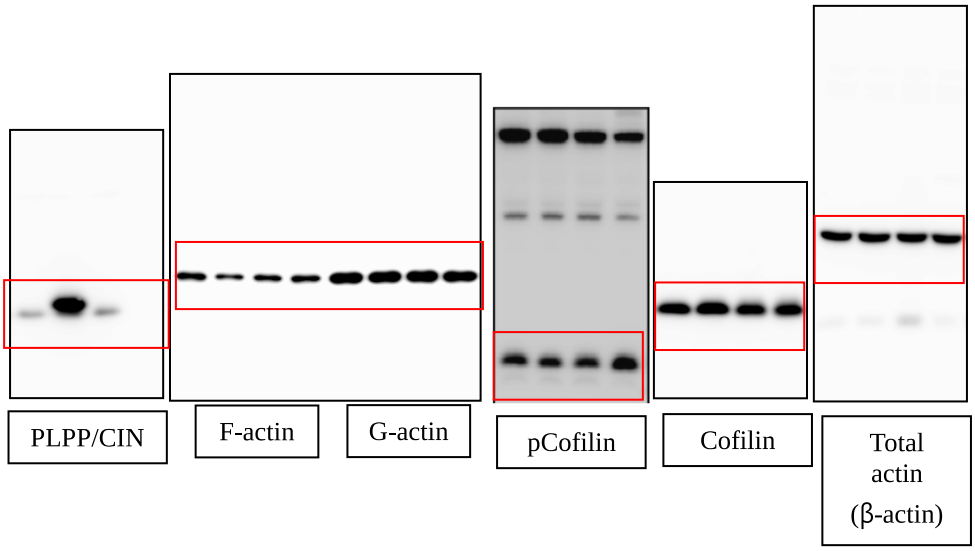

Supplementary Figure 12. Full-length gel images of western blot data in Fig. 1. The cropped parts of western blots are indicated with boxes.

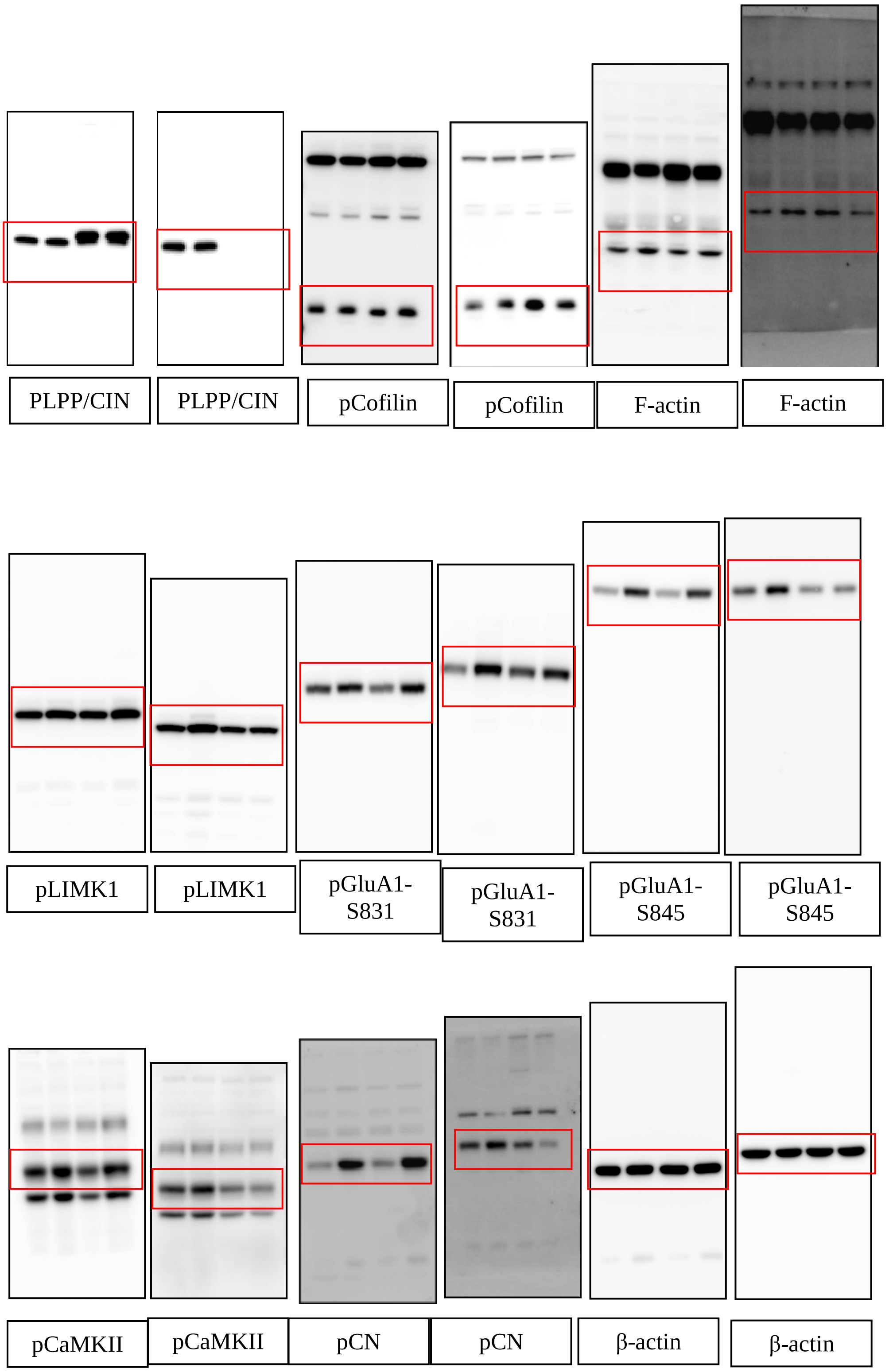

Supplementary Figure 13. Full-length gel images of western blot data in Fig. 2. The cropped parts of western blots are indicated with boxes.

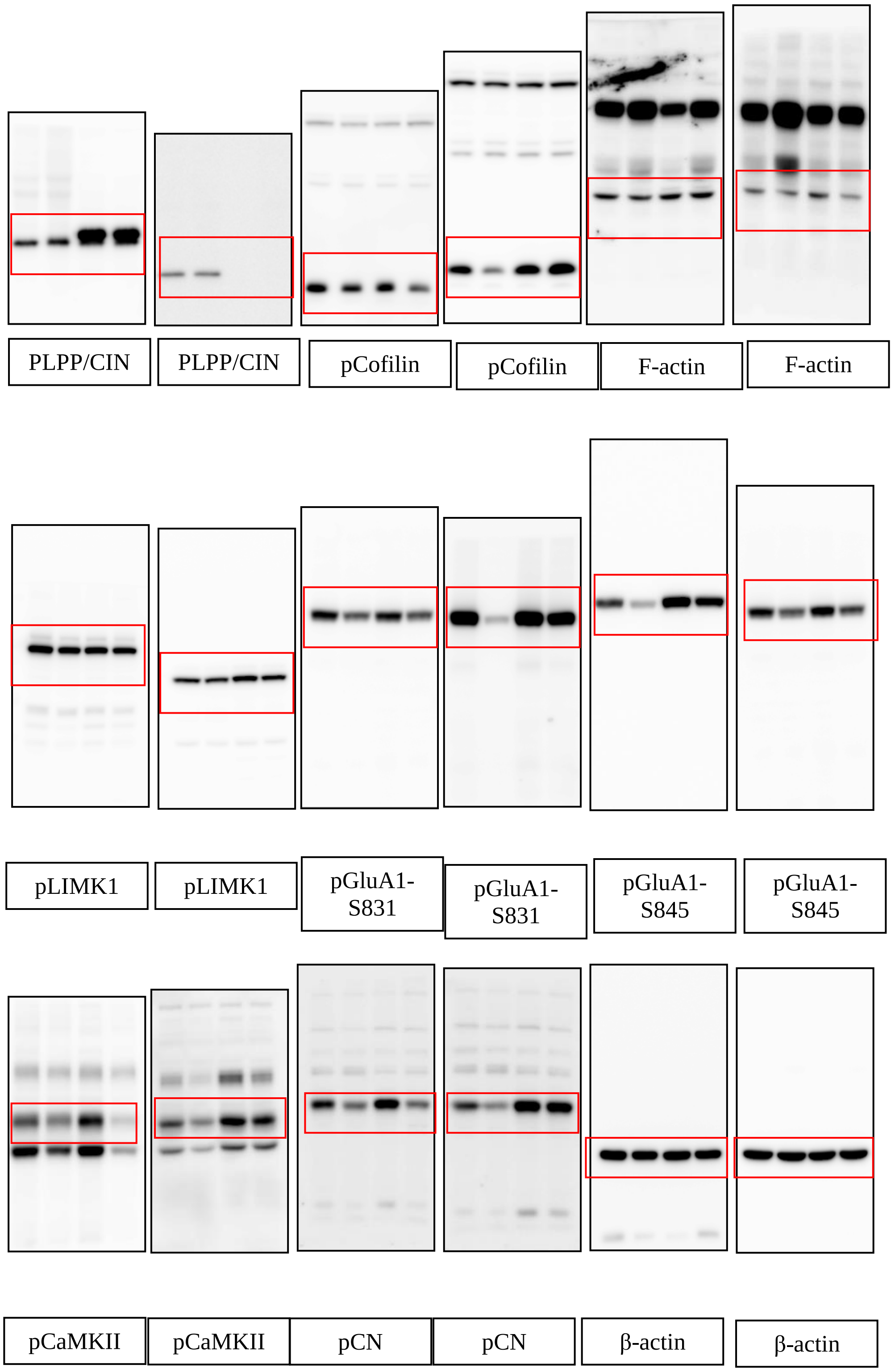

Supplementary Figure 14. Full-length gel images of western blot data in Fig. 3. The cropped parts of western blots are indicated with boxes.

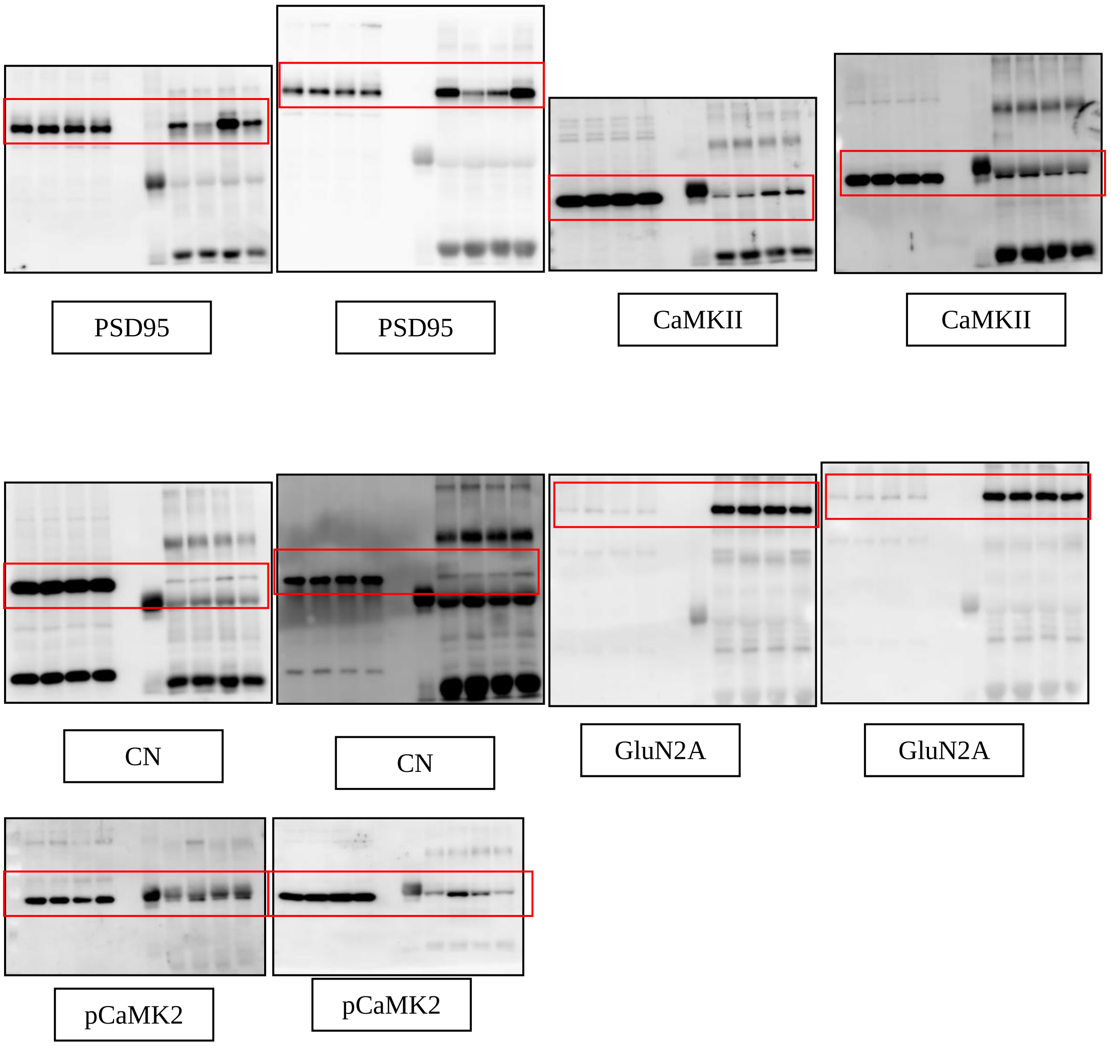

Supplementary Figure 15. Full-length gel images of western blot data in Fig. 5. The cropped parts of western blots are indicated with boxes.

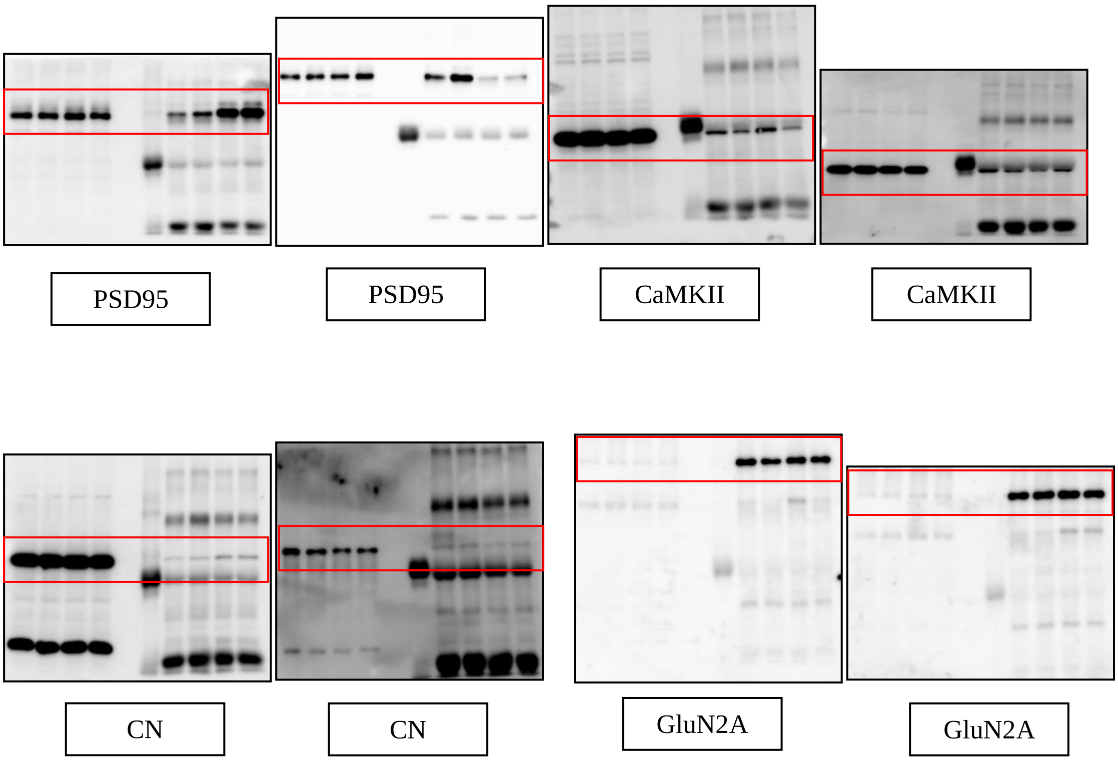

Supplementary Figure 16. Full-length gel images of western blot data in Fig. 6. The cropped parts of western blots are indicated with boxes.

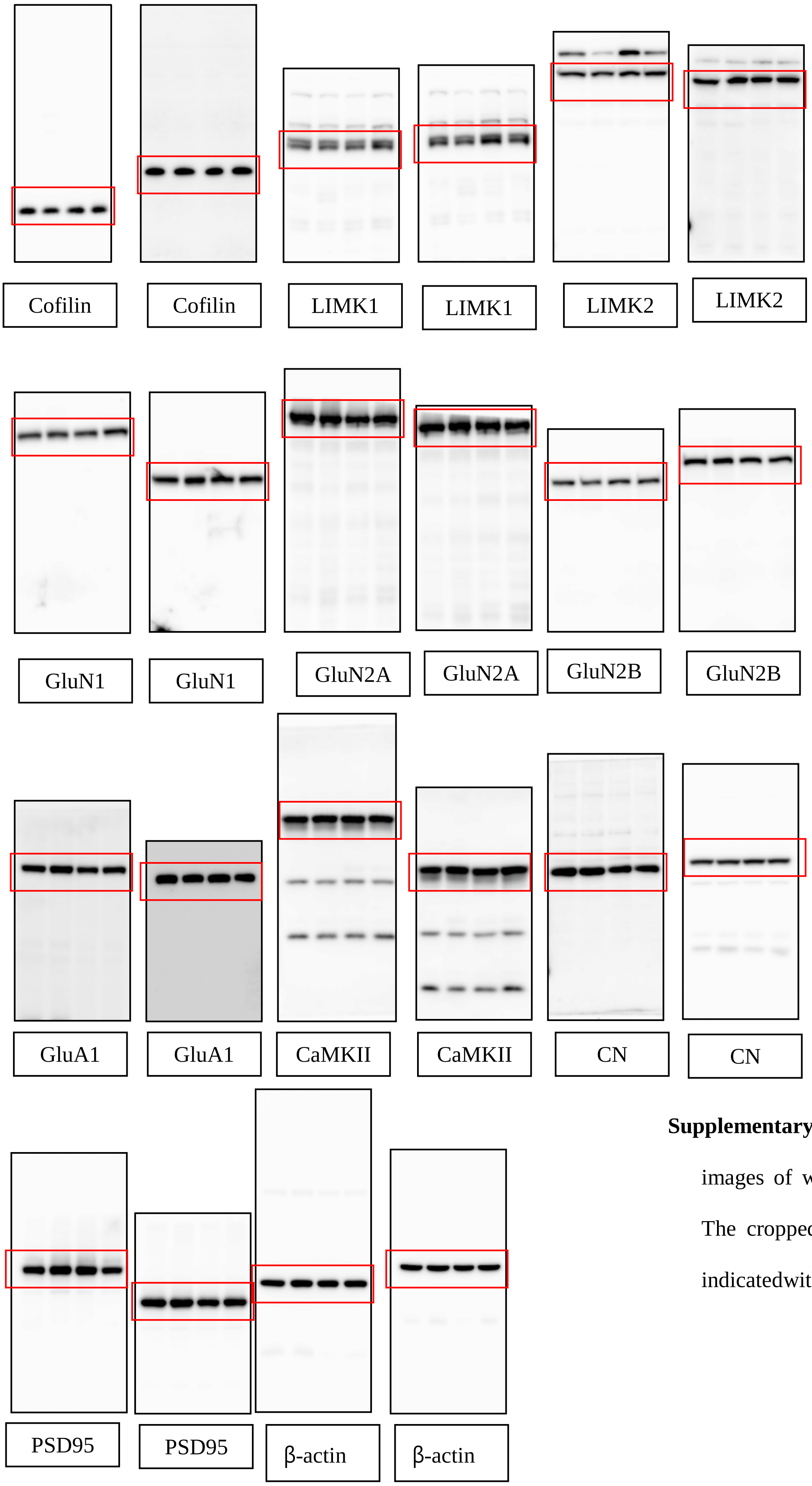

Supplementary Figure 17. Full-length gel images of western blot data in SFig. 6. The cropped parts of western blots are indicatedwith boxes.

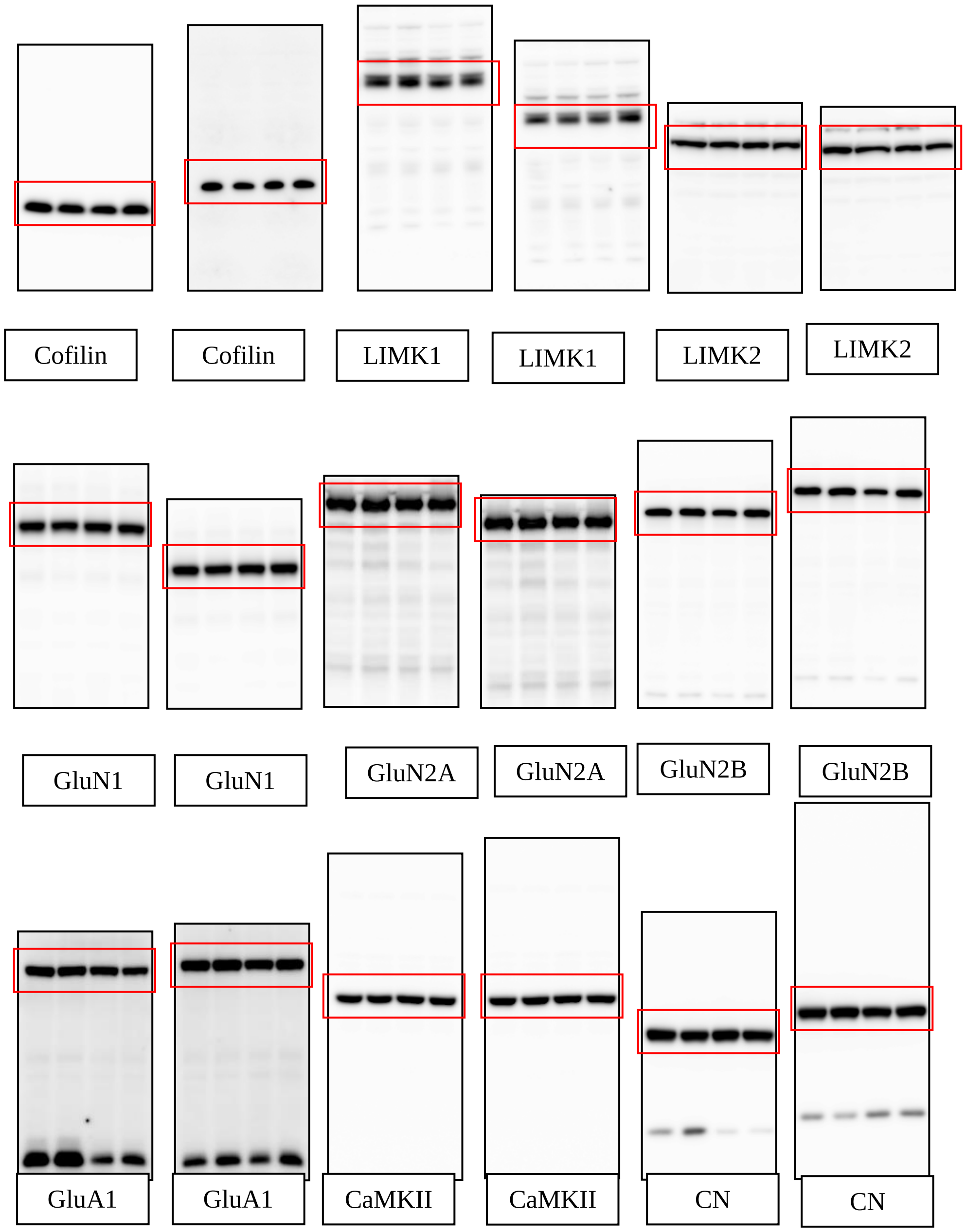

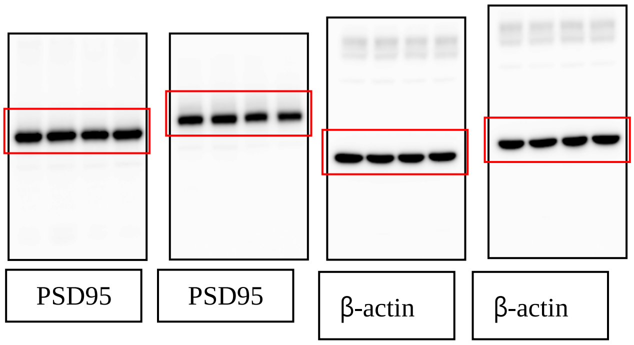

Supplementary Figure 18. Full-length gel images of western blot data in SFig. 7. The cropped parts of western blots are indicated with boxes.

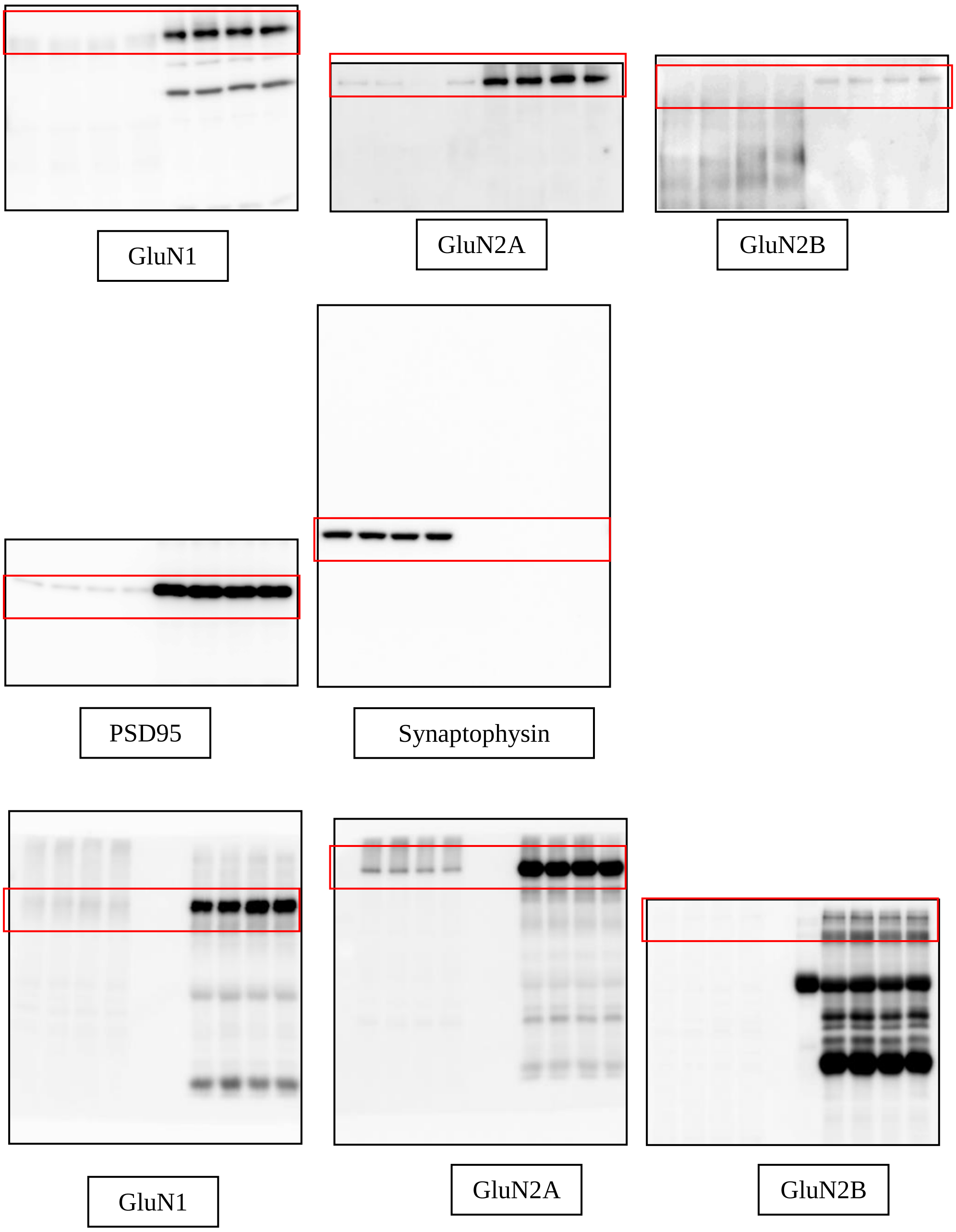

Supplementary Figure 19. Full-length gel images of western blot data in SFig. 8. The cropped parts of western blots are indicated with boxes.

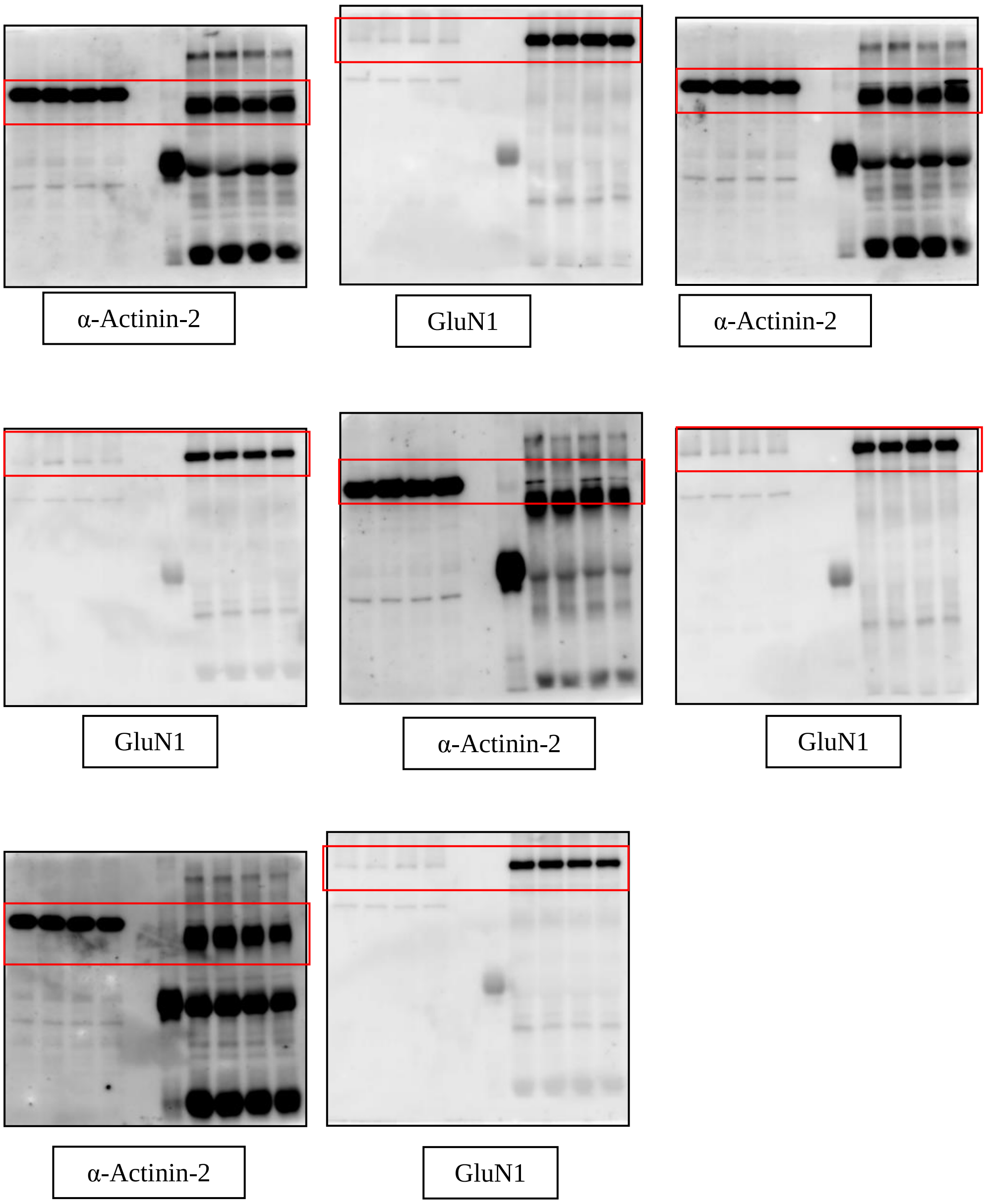

Supplementary Figure 20. Full-length gel images of western blot data in SFig. 9. The cropped parts of western blots are indicated with boxes.

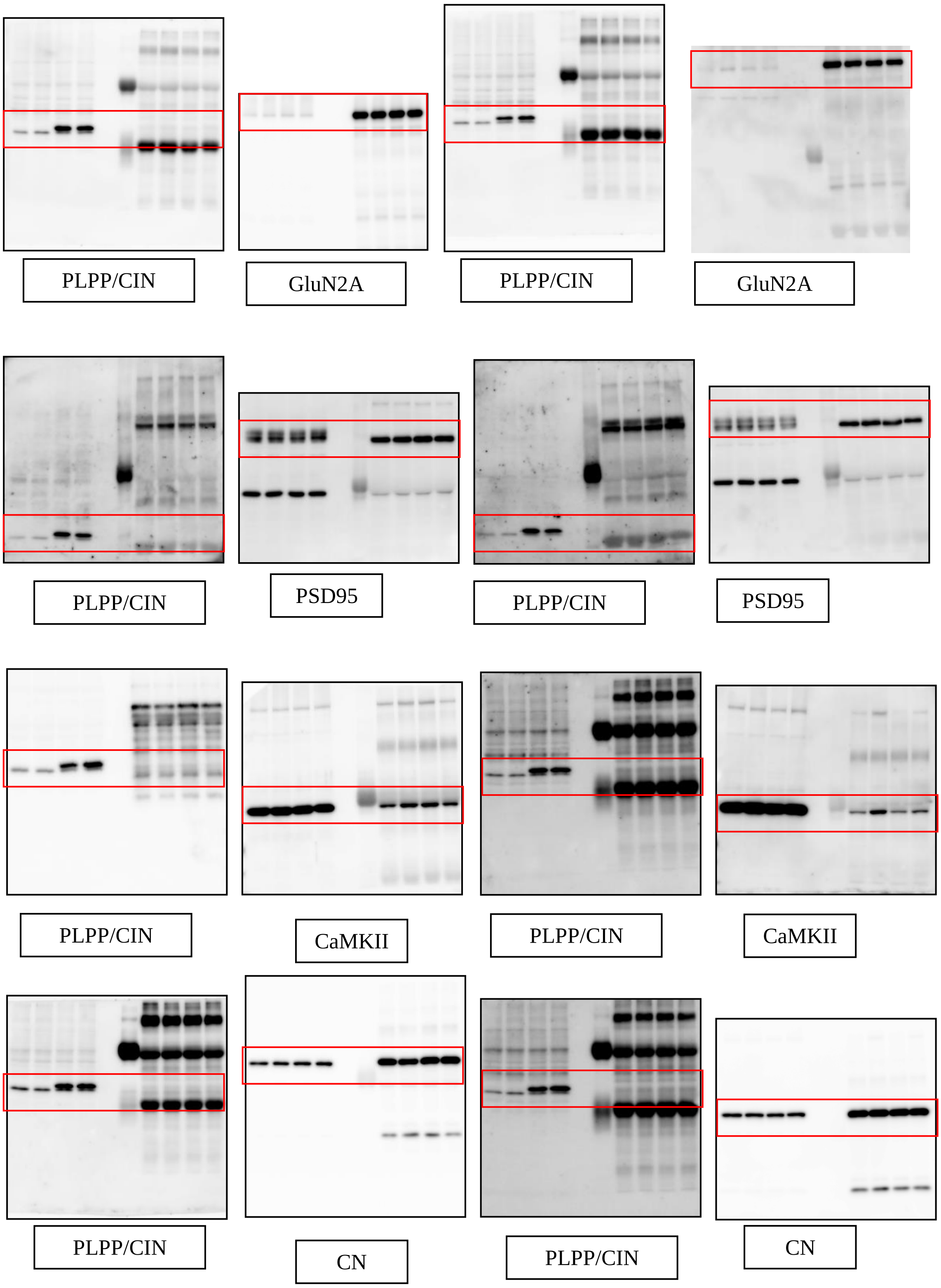

Supplementary Figure 21. Full-length gel images of western blot data in SFig. 10. The cropped parts of western blots are indicated with boxes.

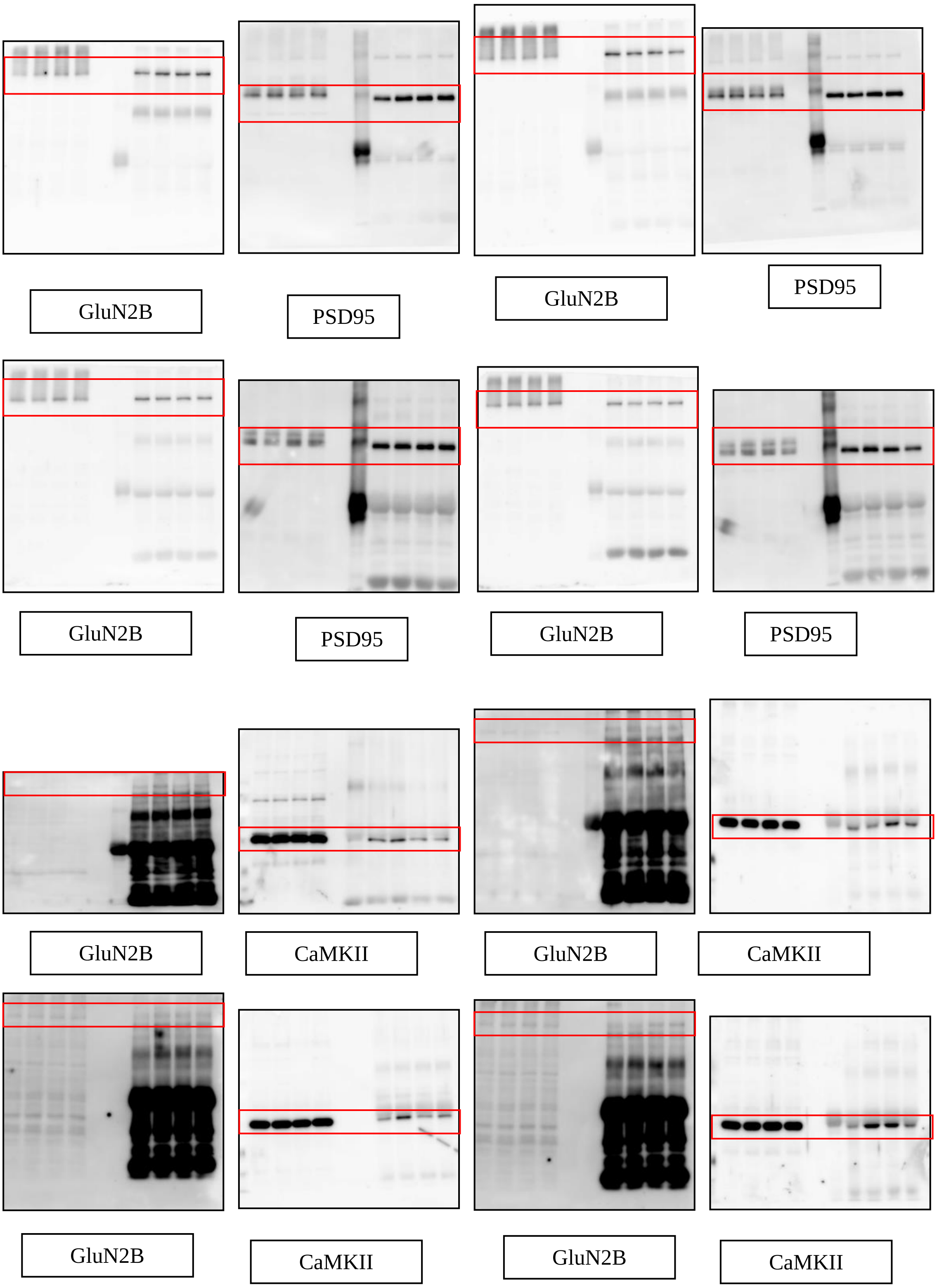

Supplementary Figure 22. Full-length gel images of western blot data in SFig. 11. The cropped parts of western blots are indicated with boxes.

Supplementary Table 1. Primary antibodies used in the present study

Antigen	Host	Manufacturer<br>(catalog number)	Dilution used
CaMKII	Rabbit	Santa Cruz (sc-13082)	1:1,000 (WB) / 1:25 (IP)
CN	Rabbit	Millipore (07-068-I)	1:1,000 (WB) / 1:50 (IP)
Cofilin	Rabbit	Sigma (C8736)	1:20,000 (WB)
F-actin	Mouse	Abcam (ab205)	1:500 (WB)
GFAP	Mouse	Millipore (Mab3402)	1:100 (IHC)
GluA1	Mouse	Synaptic system (182 011)	1:500 (WB)
LIMK1	Rabbit	Abcam (ab81046)	1:1,000 (WB)
LIMK2	Rabbit	Sigma (HPA008183)	1:1,000 (WB)
GluN1	Rabbit	Thermo Scientific (PA3-102)	1:600 (WB) / 1:100 (IP)
GluN2A	Rabbit	Thermo Scientific (OPA1-04021)	1:1,000 (WB) / 1:150 (IP)
GluN2B	Rabbit	Thermo Scientific (OPA1-04022)	1:1,000 (WB)
pCaMKII	Rabbit	Abcam (ab32678)	1:5,000 (WB)
pCN	Rabbit	Badrilla (A010-80)	1:1,000 (WB)
pCofilin	Rabbit	Abcam (ab47281)	1:1,000 (WB)
pGluA1-S831	Rabbit	Abcam (ab109464)	1:5,000 (WB)
pGluA1-S845	Rabbit	WuXi Apptec (AJ1322b)	1:1,000 (WB)
pLIMK1	Rabbit	Abcam (ab38508)	1:1,000 (WB)
PLPP/CIN	Rabbit	Sigma (HPA001099)	1:1,000 (WB) / 1:200 (IHC)
PSD95	Rabbit	Abcam (ab18258)	1:500 (WB) / 1:100 (IP)
Synaptophysin	Rabbit	Abcam (ab32127)	1:20,000 (WB)
α-actinin-2	Rabbit	Novus (EP2529Y)	1:1,000 (WB)
β-actin	Mouse	Sigma (A5316)	1:5,000(WB)

IHC, Immunohistochemistry; WB, Western blot; IP, Immunoprecipitation.
